# An innovative flow cytometry method to screen human scFv-phages selected by *in vivo* phage-display in an animal model of atherosclerosis

**DOI:** 10.1038/s41598-018-33382-2

**Published:** 2018-10-09

**Authors:** Audrey Hemadou, Jeanny Laroche-Traineau, Ségolène Antoine, Philippe Mondon, Alexandre Fontayne, Yannick Le Priol, Stéphane Claverol, Stéphane Sanchez, Martine Cerutti, Florence Ottones, Gisèle Clofent-Sanchez, Marie-Josée Jacobin-Valat

**Affiliations:** 1 CRMSB, UMR5536 CNRS, INSB, Bordeaux, 33076 France; 2 LFB Biotechnologies, department of biotherapeutic engineering Lille, Lille, 59000 France; 3 Elsevier Masson SAS, Elsevier RD solutions, Issy les Moulineaux, Issy les Moulineaux, 92130 France; 4 CGFB, Proteome pole, Bordeaux, 33076 France; 5 UPS 3044, CNRS, Saint-Christol-Lès-Alès, France

## Abstract

Atherosclerosis is a chronic, progressive inflammatory disease that may develop into vulnerable lesions leading to thrombosis. This pathology is characterized by the deposition of lipids within the arterial wall and infiltration of immune cells leading to amplification of inflammation. Nowadays there is a rising interest to assess directly the molecular and cellular components that underlie the clinical condition of stroke and myocardial infarction. Single chain fragment variable (scFv)-phages issuing from a human combinatorial library were selected on the lesions induced in a rabbit model of atherosclerosis after three rounds of *in vivo* phage display. We further implemented a high-throughput flow cytometry method on rabbit protein extracts to individually test one thousand of scFv-phages. Two hundred and nine clones were retrieved on the basis of their specificity for atherosclerotic proteins. Immunohistochemistry assays confirmed the robustness of the designed cytometry protocol. Sequencing of candidates demonstrated their high diversity in VH and VL germline usage. The large number of candidates and their diversity open the way in the discovery of new biomarkers. Here, we successfully showed the capacity of combining *in vivo* phage display and high-throughput cytometry strategies to give new insights in *in vivo* targetable up-regulated biomarkers in atherosclerosis.

## Introduction

Atherosclerosis is the main cause of death in the western world with 19 million deaths per year^1^. This chronic and systemic disease affects arteries with large and medium caliber^2^. The pathology is characterized by the build-up of lipid-rich plaques within the arterial wall, which can evolve into stable or vulnerable atheroma plaques. Stable atheroma tends to be asymptomatic unless expanding atherosclerotic lesions cause severe narrowing of the lumen, one of the consequences being ischemia. Vulnerable atheroma has more dramatic impacts due to the presence of a large lipid core covered with a thin fibrous cap at high risk of rupture and thrombi formation. These events precipitate the clinical conditions of stroke and myocardial infarction^3,4^. Nowadays, high-grade internal carotid artery stenosis (>70% luminal narrowing) is awaited before proposing endarterectomy to the patients^5^. However, the degree of luminal stenosis is not always effective in identifying high-risk patients and less than 50% occlusion in the arterial bed can lead to dramatic events. Only targeted imaging modalities able to characterize atherosclerosis at the molecular level could provide a valuable mean to diagnose vulnerability. Even though progress in understanding the atherogenic molecular basis has been made, opening up horizons for several promising novel targets such as αIIbβ3^6^ and carbonic anhydrase II (CAII)^7^, there is still a crucial need to develop clinical imaging modalities with new ligands able to target efficiently the atherogenic components^8^.

In this study, in order to assess the cellular and molecular components underlying the risk of plaque rupture in pathophysiological conditions, we proceed to an *in vivo* phage display biopanning in an animal model of atherosclerosis. Initially, *in vivo* phage display selections had been performed with peptide libraries^9–11^. We recently implemented this biotechnological approach with human scFv (single-chain fragment variable) libraries because human antibodies provide better specificity and lower immunogenicity when injected in humans. Human antibody fragments were successfully selected and shown to target new biomarkers of the pathology^7,12,13^. However, these studies used semi-synthetic human scFv libraries that typically yield antibody fragments of lower affinity. This contrasts the situation in immune libraries where the immune system proceeds to affinity maturation for specific antigens. In order to meet affinity and specificity requirements and thus a better efficacy of targeting, we switched to a fully human scFv antibody (HuAb) library with high and original complexity made of a wide naive HuAb population combined with a naturally “oriented” population of HuAb against several pathologies (autoimmune diseases, different types of cancer and cardiovascular diseases). This human library (MG-Umab) was further hyper-diversified by random mutagenesis (patent N° WO0242311, WO2007137616)^14,15^ in order to generate 3.4 billion clones with 94% of them encoding full-length variable genes. Here, we described the successful selection of scFv-phages obtained from this fully human library by three rounds of *in vivo* phage display biopanning in atherosclerotic New Zealand White (NZW) rabbits combined with high-throughput flow cytometry screening. This method is highly suitable to test thousands of *in vivo* selected scFv-phages. Individually selected phage-antibodies whose specificity was confirmed by immunohistochemistry (IHC) will be of interest in the discovery of novel targets for a better understanding of the pathogenesis of atherosclerosis and the development of accurate diagnosis tools.

## Results

**Figure 1:**
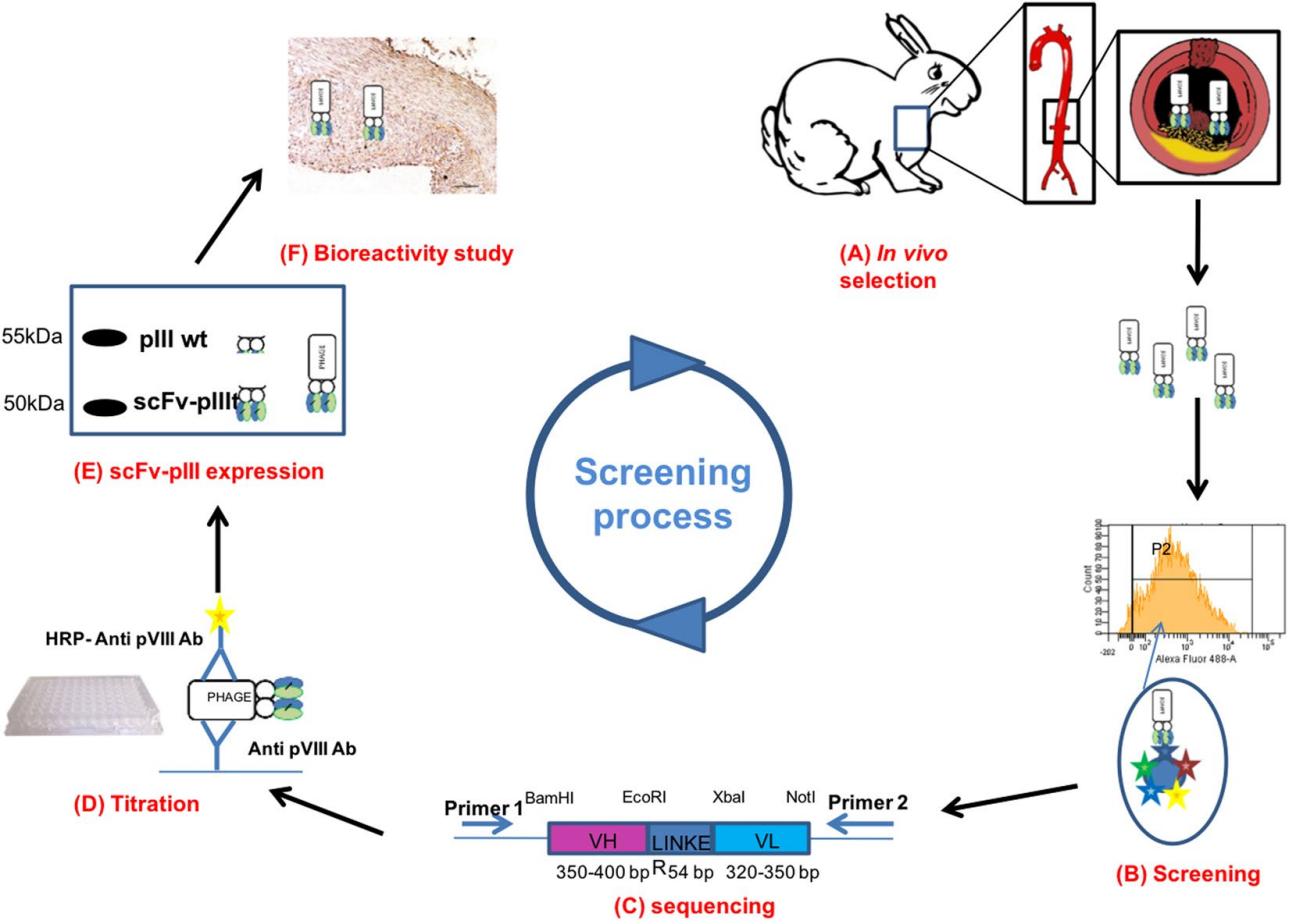
Representative scheme of the developing process of antibodies able to recognize atheroma plaques. (**A**) Human scFv-phages were injected in atherosclerotic NZW rabbits for *in vivo* recognition of cellular and molecular atherogenic components in the aorta. After *in vivo* biopanning, scFv-phages were retrieved from rabbit aorta. (**B**) Flow cytometry was used to individually screen scFv-phages issuing from *in vivo* phage-display against atherosclerotic proteins. (**C**) The clones were sequenced to evaluate the integrity of the two V-Regions. (**D**) Quantitative ELISA was used to quantify individual scFv-phage clones produced in 96-well culture plates. (**E**) scFv-pIII protein expression was controlled by Western Blotting. (**F**) The bioreactivity of human scFv-phage candidates was validated on atherosclerotic sections by immunohistochemistry.

The successive selection steps to identify scFv-phage candidates able to target cellular and molecular atherogenic components are summarized in Fig. 1. The whole strategy combines two methodologies: *in vivo* phage display and high-throughput flow cytometry. Briefly, scFv-phages issued from a fully human library were exposed to atherogenic cellular and molecular components of rabbit aorta by an *in vivo* approach. Individual clones isolated during this *in vivo* selection were then screened by flow cytometry analysis against balloon injured aortas of hypercholesterolemic NZW rabbit (BIAHR) proteins. After quantification of scFv-phages by ELISA, western blotting was performed for further control of scFv-pIII expression. Selected hits were validated by IHC for bioreactivity assays.

### *In vivo* phage display selection of human scFvs in a complex animal model of atherosclerosis

**Figure 2:**
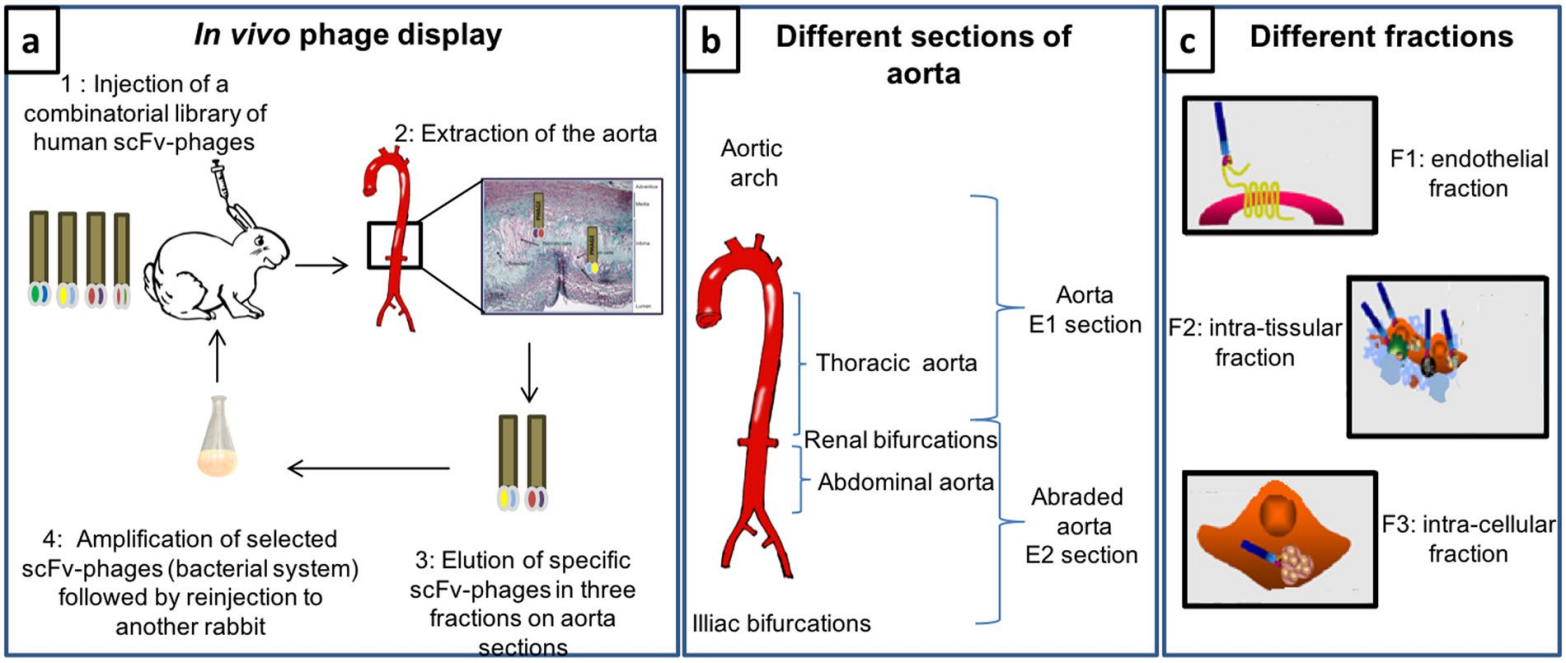
Schematic representation of the *in vivo* phage display procedure. (**a**) Three rounds of biopanning were performed in atherosclerotic NZW rabbits. ScFv-phages were eluted from different fractions of the extracted aorta. (**b**) Fractions issued from two sections of the aorta, E1 and E2 sections. E1 section included the aortic arch and the thoracic area and the E2 section corresponded to renal and abdominal areas which were abraded by balloon injury. (**c**) F1: recovered scFv-phages from endothelial fraction, F2: recovered scFv-phages from intra-tissular fraction and F3: recovered scFv-phages from intra-cellular fraction.

ScFv-phages isolated from the lesions (from the aortic arch to the thoracic aorta and from the renal to the iliac bifurcations) constitute the round 1-selected scFv-phage library (Fig. 2a,b). Iterative cycles of *in vivo* selection and amplification (additional round 2 and round 3) in subsequent NZW rabbits were performed to enrich for more specific candidates (Fig. 2a). Furthermore, in order not to restrict the analysis only on sites directly in contact with blood, scFv-phages emigrating within the intima were recovered. Homing scFv-phages were thus isolated not only from the injured endothelium (F1 fraction) but also from the underlying lesional tissue (F2 fraction) and within the cells from the intima (F3 fraction) (Fig. 2c). The number of scFv-phages recovered (cfu) was normalized for an input of 10^12^ cfu (in order to compare the iterative cycles of *in vivo* biopanning) and calculated per mg of tissue. The number of recovered scFv-phages from F2 and F3 fractions increased after the third round of selection compared to the first round (Supplementary Fig. S1). The implemented technology has thus led to the *in vivo* selection of scFv-phages able to reach subjacent lesional tissue layers and internalized within cells invading the intima.

### High-throughput biological screening

One of the drawbacks of *in vivo* selections is the screening strategy of the selected clones. Indeed, the antigen is not identified and that limits the use of classic ELISA assays where purified antigens are usually coated on plates at a quite high concentration (10 µg/mL). We propose as an alternative a high-throughput flow cytometry plaque-assay using soluble tissue extracts coupled to magnetic beads supposed to be more sensitive compared to ELISA with less biological material needed. Indeed, only 200 ng of proteins (see Methods section) are used per well to screen individual clones by flow cytometry, meaning 5 fold less than for an ELISA assay. Moreover, high-throughput screening should allow testing thousands of clones obtained from *in vivo* biopanning.

#### Pathway Studio analysis of proteins differentially represented in atherosclerotic NZW rabbits

It was firstly necessary to assess the diversity and over-representativity of proteins in the BIAHR extracts. Because of the lack of data in the rabbit database, the protein extraction composition was compared to the human database. The ratios of proteins between BIAHR and Healthy (H) extracts were calculated from the normal abundance and then imported and studied by an international software, Pathway Studio.

**Figure 3:**
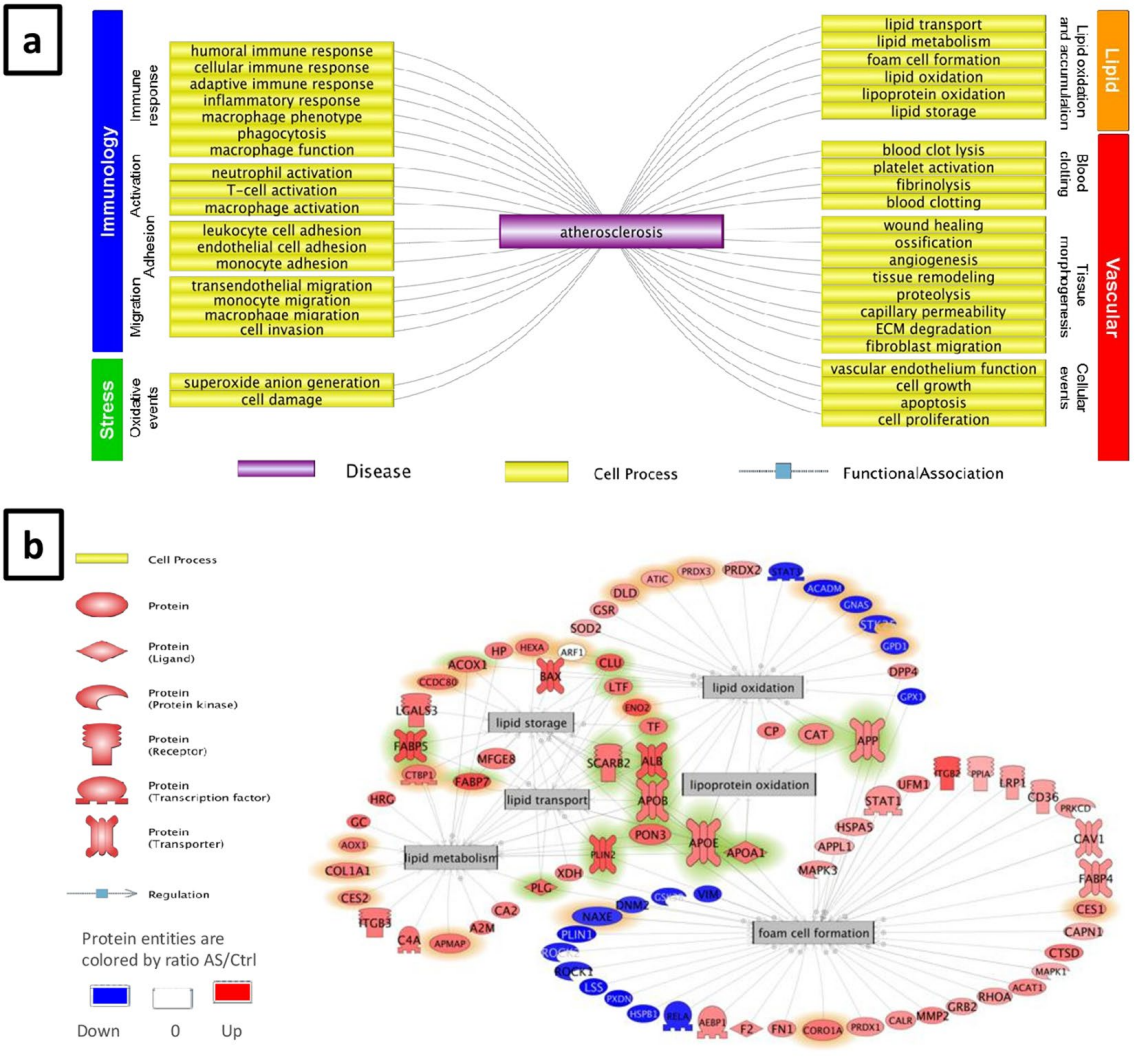
Enriched cell-process subnetworks in aorta samples from atherosclerotic NZW rabbits compared to control NZW rabbits. (**a**) Analysis of cell processes related to overexpressed proteins in atherosclerotic plaque of hypercholesterolemic NZW rabbits. A representative selection of the 178 enriched cell processes reported in Supplementary Table S1 is shown. Cell processes not linked to atherosclerosis in Pathway Studio or belonging to the miscellaneous category, and some redundant cell processes in the other categories are not displayed. Enriched cell processes are regulated by multiple proteins with differential abundance in aorta protein extracts of atherosclerotic and healthy NZW rabbits. Cell processes are grouped in 4 categories: immune-, vascular-, stress- and lipid-relatedevents. All links are supported by biological findings from scientific literature. (**b**) Enriched subnetworks related to lipid cell processes. Protein entities colorized in red and blue are respectively down- and over-represented in aorta protein extracts of atherosclerotic compared to healthy NZW rabbits. Proteins highlighted in green are co-regulating in 3 or more of the 6 cell processes. Proteins highlighted in orange are not connected to atherosclerosis disease entity in Pathway Studio. All links are supported by biological findings from scientific literature. Variation of entity name police size does not have any biological meaning.

The proteomic profile of balloon-injured aortas in hypercholesterolemic NZW rabbits reveals atheromatous characteristics: The study of the data issued from the proteomic analysis was performed following two approaches, involving cell process subnetwork analysis in Pathway Studio, one considering the entire data set using the GSEA-based (SNEA) test^16^, the other focusing on the top 100 over-represented proteins analysed by Fisher’s based (FSNE) analysis^17^. In a first step, the analysis showed that the over-represented proteins in BIAHR compared to H aortas are implicated in a large diversity of cell processes that have been related to either immunological-, vascular-, stress- or lipid-related events (Supplementary Table S1). In a second step, the question was: *what cell processes are linked to atherosclerosis?* Almost half of the top cell processes found by GSEA and FSNE tests were found to be functionally associated with atherosclerosis in the Pathway Studio knowledge base of millions of biological findings from peer-reviewed literature (Supplementary Table S1). A representative selection of those cell processes helped showing that most of these events were described to contribute to the natural history of atherosclerosis^2,18^ (Fig. 3). These data gave us confidence in the composition of our BIAHR extracts. Given the involvement of the immune response in the context of atherogenesis, it was not surprising that immunological cell processes were over-represented (81/178) and many of them related to the activation, migration or adhesion of immune cells. Interestingly, some of enriched cell processes were linked to lipids (18/178), stress (2/178) or events implying vessels (42/178) such as blood clotting, tissue morphogenesis and more general cell processes related to cell growth, cell proliferation and apoptosis (Fig. 3a, supplementary Table S1).

**Figure 4:**
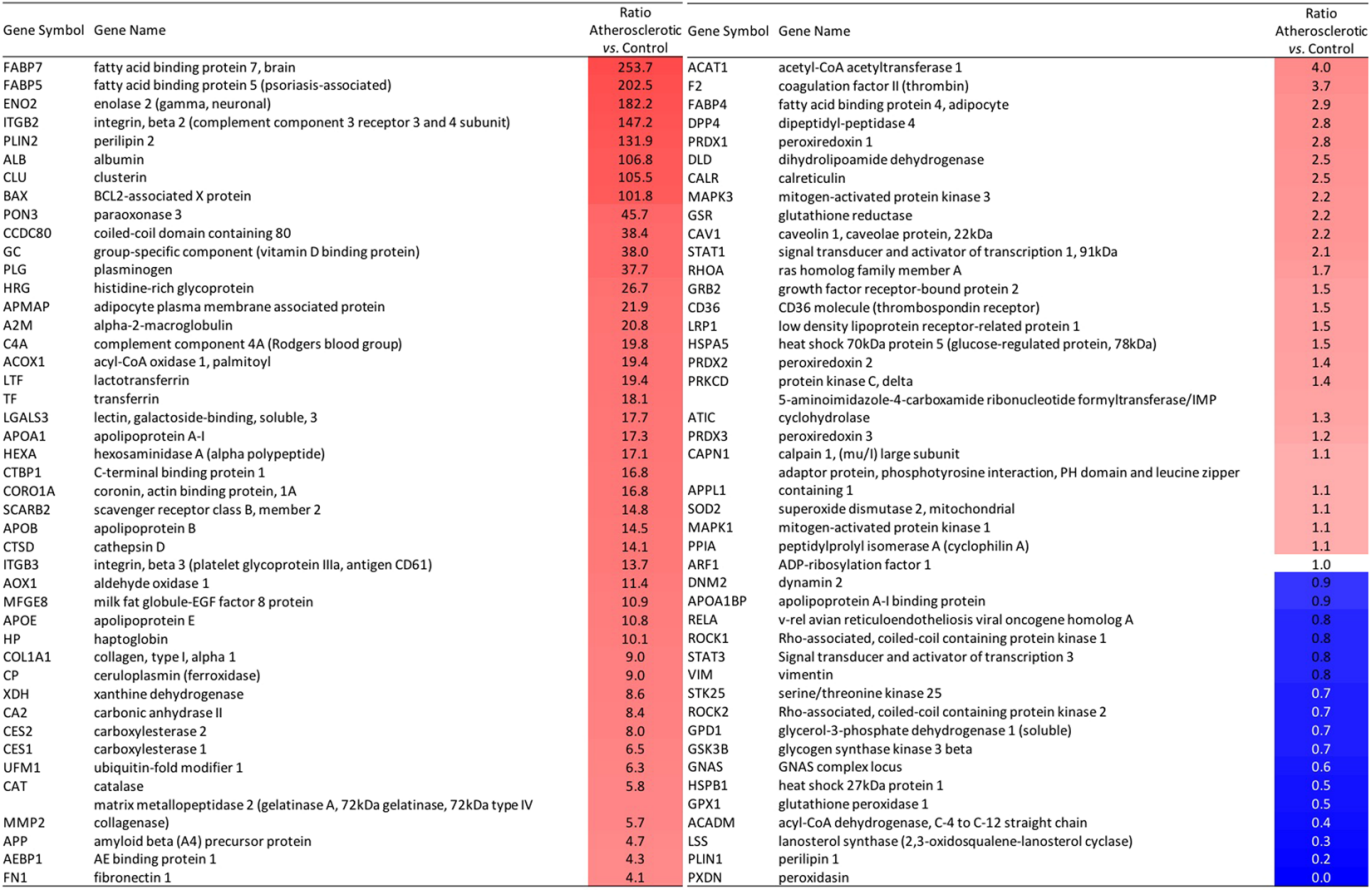
Abundance of proteins belonging to lipid-related cell process subnetworks enriched in BIAHR rabbits compared to aorta of H rabbits. Protein entities colorized in red and blue are respectively up- and down-regulated in aorta samples of atherosclerotic compared to control NZW rabbits. Ratio values are reported.

Zoom on proteins involved in lipid-related cell processes: We chose to focus on lipid-related processes because of the high involvement of lipids in atherogenesis and to highlight more specifically foam cell formation, which is a major characteristic of vulnerable atheroma plaques. The protein subnetwork regulating lipid oxidization (Fig. 3b) was found significantly enriched in both Fisher’s (p = 1.7 × 10^−9^) and GSEA enrichment test (p = 5.7 × 10^−3^). Protein networks regulating lipid storage, lipid transport and lipid metabolism were found enriched with FSNE test only (p = 6.9 × 10^−9^, p = 3.8 × 10^−10^ and p = 6.3 × 10^−15^ respectively). The one regulating foam cell formation was found significantly enriched only in GSEA enrichment test (GSEA p = 7.1 × 10^−3^) (supplementary Table S1). It was shown here that some proteins are co-regulating multiple lipid-related cell processes. Some of them are well known to be increased in atherosclerosis such as apolipoprotein E (ratio = 10.82), ApoB (ratio = 14.53) and APOA1 (ratio = 17.33) as well as paraoxonase 3 (PON3) (ratio = 45.74)^19,20^ (supplementary Table S2). Some highly overexpressed proteins in BIAHR compared to H were less known as being involved in atheroslerosis. It includes Fatty acid-binding protein 7 (FABP7) (ratio = 254.7), Enolase 2 (ENO2) (ratio = 182.2) and Acyl-coenzyme A oxidase 1 (ACAO1) (ratio = 19.4). The most over-represented proteins in BIAHR compared to H involved in foam cell formation were the integrin β2 (ratio = 147.2), Perilipin 2 (PLIN2) (ratio = 132), Plasminogen (PLG) (ratio = 37.7), Coronin, actin-binding protein 1 A (CORO1A) (ratio = 16.8), Cathepsine D (CTSD) (ratio = 14.1), Xanthine deshydrogenase, (XDH) (ratio = 8.6), Carboxylesterase1 (CES1) (ratio = 6.5), Ubiquitin fold modifier 1 (UFM1) (ratio = 6.3), Catalase (CAT) (ratio = 5.8), Matrix metalloprotease 2 (MMP2) (ratio = 5.65) (Fig. 4). All these results highlighted over-represented proteins that may be considered as new biomarkers of atherogenesis, potentially targeted by human antibodies issued from our *in vivo* selection. Other proteins are down-represented in the foam cell formation sub-network, such as Rho associated protein kinase (ROCK2) (ratio = 0.7), Glycogen synthase kinase 3 beta (GSK3B) (ratio = 0.7), Heat shock 27 kDa protein 1 (HSPB1) (ratio = 0.5), Glutathione peroxidase 1 (GPX1) (ratio = 0.5), Lanosterol synthase (LSS) (ratio = 0.3) and Perilipin 1 (PLIN1) (ratio = 0.2) (Fig.4).

#### Evaluation of the coupling of BIAHR proteins onto the beads

**Figure 5:**
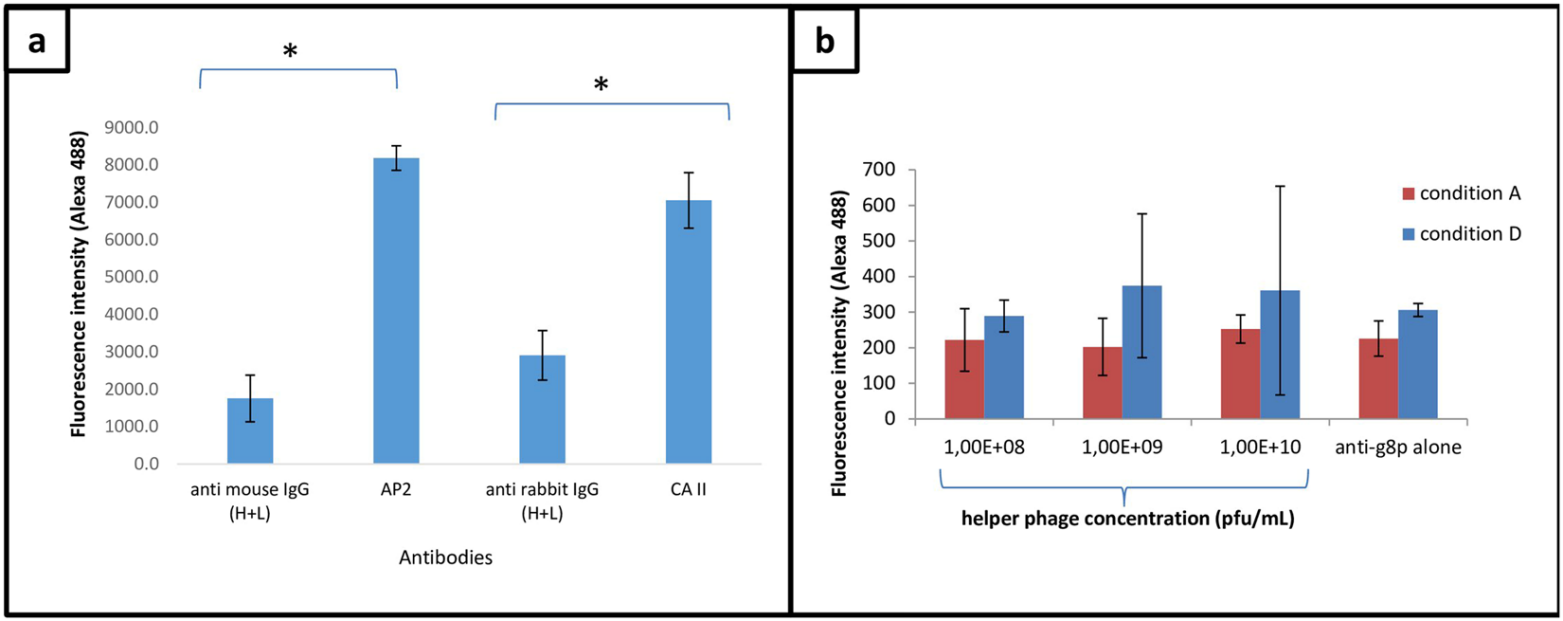
Implementation of the flow cytometry strategy. (**a**) Control antibodies were tested by flow cytometry to guarantee the quality of the coupling strategy. AP2: mouse anti-αIIbβ3 antibody, CAII: rabbit anti-carbonic anhydrase II (CAII) antibody (Abcam) and RAM11: murine RAM11 antibody (anti-CD68). Secondary antibodies are Alexa 488 F(ab’)_2_ anti-mouse and Alexa 488 F(ab’)_2_ anti-rabbit. (**b**) Fluorescence intensity (geomean) of hyperphage particles from 10^8^ to 10^10^ cfu/mL in A and D conditions.

For an efficient screening of scFv-phages by flow cytometry, it is important to know the rate of coupling of the protein extracts to the magnetic beads. Thus, the three batches of coated protein beads were analysed by SDS-PAGE electrophoresis. In electrophoresis conditions, only the uncoupled fractions of protein-beads preparations were able to migrate through the gel matrix, the beads coated with BIAHR proteins being stacked on top of the gel. In Fig. S2 (given in supplementary data), the signal obtained with the uncoupled fraction is identical between the three batches and in the same range as this obtained with 8 to 10 µg of the whole extract loaded on wells. The coupling rate was estimated by ImageJ analysis at approximatively 80% (Supplementary Fig. S2). We designed a first flow cytometry assay (Fig. 5a) to evaluate, after coating, the integrity and the folding of the proteins by using AP2 and CAII murine antibodies. These antibodies were chosen for their well-known capacity to bind proteins present in the atherosclerotic extract with different ratio BIAHR on H proteins (13.69 for integrin beta3 and 8.37 for carbonic anhydrase II) (see the ratio of proteins in Supplementary Table S2). The results obtained assessed the quality of the coupling and accredited the whole flow cytometry strategy. Indeed, as shown in Fig. 5a, the increase of the fluorescence intensities of AP2 and CAII over the secondary antibodies confirmed their binding on their respective targets (αIIbβ3 integrin and the carbonic anhydrase II enzyme).

### Determination of accurate screening conditions

The phage display technology has the inconvenience to amplify a large amount of recombinant phages that do not display the scFv fragment because they are more able to infect bacteria with a high efficacy (95% of phages without scFv expression have been estimated by using M13KO7 helper phage). The hyperphage used in this study, in contrast to M13KO7 helper phage, was designed to force the *E*. *coli* cell to use the pIII portion of the antibody fusion protein for packaging. However, a significant amount of phages still not display scFv fragments (Fig. S4). These phages may bring about non-specific binding during the screening process, which is necessary to reduce. It was thus mandatory to set up the conditions for flow cytometry analysis to minimize the non-specific binding of hyperphage particles on coated beads. For that, we used different concentrations of hyperphage particles from 10^8^ to 10^10^ pfu/mL in accordance to the range of scFv-phage production classically obtained in masterblock cultures.

As shown in Fig. 5b, condition D, the fluorescence intensity increases when using hyperphages concentrations from 10^9^ to 10^10^ pfu/mL, highlighting a non-specific binding linked to the concentration itself. Moreover, a significant increase in variation has to be noted, depicted by the error bars. Therefore, optimization is necessary to remove variation. The non-specific binding could be explained by the lack of centrifugation and washing after the addition of anti-pVIII antibody (Table 1). We thus introduced these steps in condition C. The problem remained with the concentration of 10^10^ pfu/mL giving a high background signal (data not shown). We tested another strategy by removing centrifugation and washing steps after overnight hyperphages/proteins incubation, before anti-pVIII antibody incubation (conditions A and B). Surprisingly, this notably reduced the background signal. An hypothesis could be that the excess of anti-pVIII antibody may be washed out by hyperphage particles not withdrawn between overnight hyperphages/proteins incubation and addition of anti-pVIII antibody. This wash-out may thus reduce the recognition of the hyperphage without scFv bound non-specifically on atheromatous proteins coated on beads (Supplementary Fig. S3). We finally chose condition A in which only one centrifugation and one washing were performed to remove the residual anti-pVIII antibody and anti-pVIII antibody bound to hyperphage before addition of the secondary antibody. To conclude, in condition A, the variation is minimal whatever the concentration of hyperphage particles used, showing a minimal non-specific binding with minimal variation within each experiment. This is an important point considering that at the time of the flow cytometry experiment the rate of production of each clone could not be determined. Indeed, to be in line with a high-throughput strategy, it is impossible to quantify by ELISA each clone of a 96 deep-well culture plate. ELISA experiment will only be conducted for flow cytometry selected hits for further validation steps.

**Table 1:** Summary of the different conditions tested by Flow cytometry analysis to reduce the non-specific binding of hyperphage particles.

Conditions	Beads/phages incubation	Centrifugation	Washing	Anti pVIII incubation	Centrifugation	Washing	Secondary antibody incubation
Condition A	+	−	−	+	+	+	+
Condition B	+	−	−	+	+	−	+
Condition C	+	+	+	+	+	+	+
Condition D	+	+	−	+	−	−	+

The addition or not of the different steps is indicated by (+) or (−).

### Screening of phage antibodies by flow cytometry on BIAHR proteins

**Figure 6:**
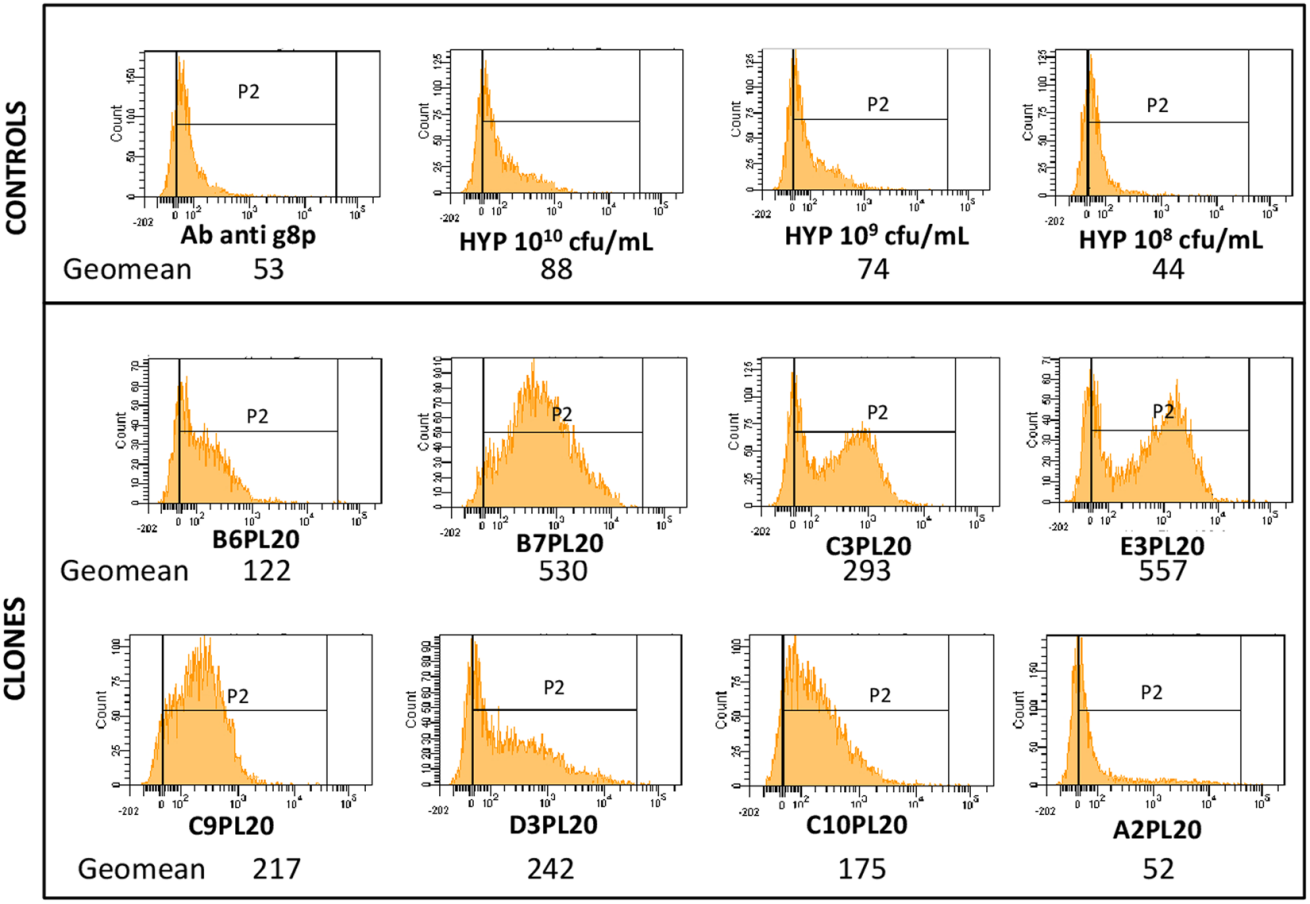
Flow cytometry analysis typical experiment of atherosclerotic protein–binding scFv-phages. The number of coated beads (counts:Y-axis) is given as a function of the fluorescence intensity of scFv-phage staining (Alexa Fluor 488:X-axis). Protein binding was detected by mouse anti-pVIII antibody and Alexa Fluor 488-conjugated anti mouse antibody. The geomean of eight clones is given as a measure of the fluorescent intensity. The positivity is defined by at least 2-fold over the averaged fluorescence intensity of hyperphage particles at different concentrations.

In order to identify a panel of individual human scFv-phages specific for proteins over-represented in atheroma plaques, a high-throughput *in vitro* screening step against an atheroma protein extract is required (supplementary Fig. S5). To that end, eight hundred sixty four clones from the third round of biopanning were produced from F1, F2 and F3 fractions in 96 deep-well masterblocks (PL19 to PL27) (Supplementary Table S3). They were tested, by flow cytometry following previously implemented condition A, for their reactivity against atheroma protein extracts. Overall, 209 clones (24%) reacted positively with atherosclerotic proteins by binding about 2 fold or more over the background signal produced by hyperphage. The threshold was arbitrary fixed by the geomean obtained on all positive fluorescent events (>0) defined as a P2 gate. Figure 6 shows typical examples of fluorescent intensities of eight positive and negative clones from PL20 measured by their geomean value calculated on this P2 gate. We can see that a good correlation was obtained between this theoretical definition of positivity by geomean comparison and the switch of the fluorescence towards higher values for reactive recombinant phages. The peaks were quite different from each other, considering the differences in terms of representativity of the target on the beads as well as productivity of each scFv-phage clone. Here, the panel of fluorescence histograms summarizes the diversity of reactivity of clones, some of them being categorized as highly positive and others with positivity just above the threshold. Is also illustrated a clone which was considered as negative. For the sake of clarity, only the results of one of the three batches are shown, knowing that the results within the three batches are always highly reproducible in each experiment. It is important to underline that this high-throughput flow cytometry strategy is a pre-screening approach, which needs further validations of the candidates by (1) sequencing the scFv fragments to control the integrity of VH and VL genes (2.5 section) and (2) evaluating the reactivity of scFv-phage clones within the context of atheroma burden by immunohistochemistry (IHC) assays on atherosclerotic NZW rabbit sections (2.7 section). Before IHC experiments, the quantification of the scFv-phages production rate in masterblocks and the accurate display of scFv-pIII on top of the hyperphage determined by ELISA and Western blotting respectively, are necessary (2.6 section). Some of the clones that were found non-reactive in flow cytometry were also introduced in the assays as controls.

### ScFv antibody sequencing and bioinformatic analysis

In order to control the integrity of variable VH and VL genes of the scFv-phage clones pre-selected on atheromatous proteins by flow cytometry and to evaluate their germline and juntional diversity, their whole scFv genes were sequenced and analysed by IMGT/V-QUEST information system using the new option “Analysis of single chain Fragment variable (scFv)”^21^ (Supplementary Methods S1). Moreover, this analysis allowed us to check the functionality of the whole scFv with in frame CDR3H and CDR3L junctions (Supplementary Table S3). Among the 209 clones selected by flow cytometry, 68% of them have a complete sequence with 42% without stop codons. Analyses of the variable germlines and CDR3 junctions showed a high diversity among the clones, excluding a potential biased clone enrichment (Supplementary Table S3). In conclusion, the scFv sequence analysis underlies a potential diversity of paratopes and unbiased clone abundance in the round 3 of *in vivo* phage selection, highlighting a potential diversity in targeted biomarkers present in the pool of atheromatous proteins.

### Quantification of phage production and expression of g3p-scFv after hyperphage superinfection

We recall that, in spite of the use of hyperphage, during expression of the scFv-pIII, the supernatant contains both wild-type and recombinant phages. So, in order to evaluate the display of pIII-scFv on top of the recombinant phages, selected clones were analysed by western blotting after ELISA quantification of the whole production (wild-type and recombinant phages) (Supplementary Methods S2). The concentration of eight clones produced individually was estimated by ELISA in regards to a range of hyperphage particles (10^8^ to 10^11^ pfu/mL). For these clones, the concentrations varied between 5.10^9^ and 10^10^ pfu/mL. These concentrations higher than the loading threshold in western blotting (around 10^8^ phages) allowed their analysis to detect the scFv-pIII expression. Figure S4 (given in Supplementary Information) shows that all the clones expressed the wild-type pIII and the pIII-scFv protein. In contrast to what is described in the literature, the band representing the scFv-pIII is lower than what is observed for the wild-type pIII. This is explained by the use, for the building of the scFv-library, of a plasmid, which contains a truncated pIII gene (pIIIt) (patent WO2007137616). We can notice that there is a great variability in expression level of the scFv-pIII protein between the different clones. Indeed, C9PL20, D3PL20, C10PL20 and B6PL20 presented a stronger expression of the scFv-pIII than E3PL20, C3PL20, B7PL20 and A2PL20. This is especially true for E3PL20 for which a tiny band at the scFv-pIII level is observed. We can also notice that the chosen negative clone A2PL20 showed a moderate expression of the scFv-pIII protein. All along the study, for a given clone, we observed from batch to batch differences in production level (Supplementary Fig. S4). In spite of unequal expression profiles, we decided to pursue with all the clones to determine their bioreactivity on atherosclerotic sections, keeping in mind that the reactivity may not only be impacted by unequal representativeness of the target on coated beads but also by the expression level of scFv on top of recombinant phages.

### Evaluation of the selected scFv-phages reactivity on atherosclerotic NZW rabbit tissue sections

**Figure 7:**
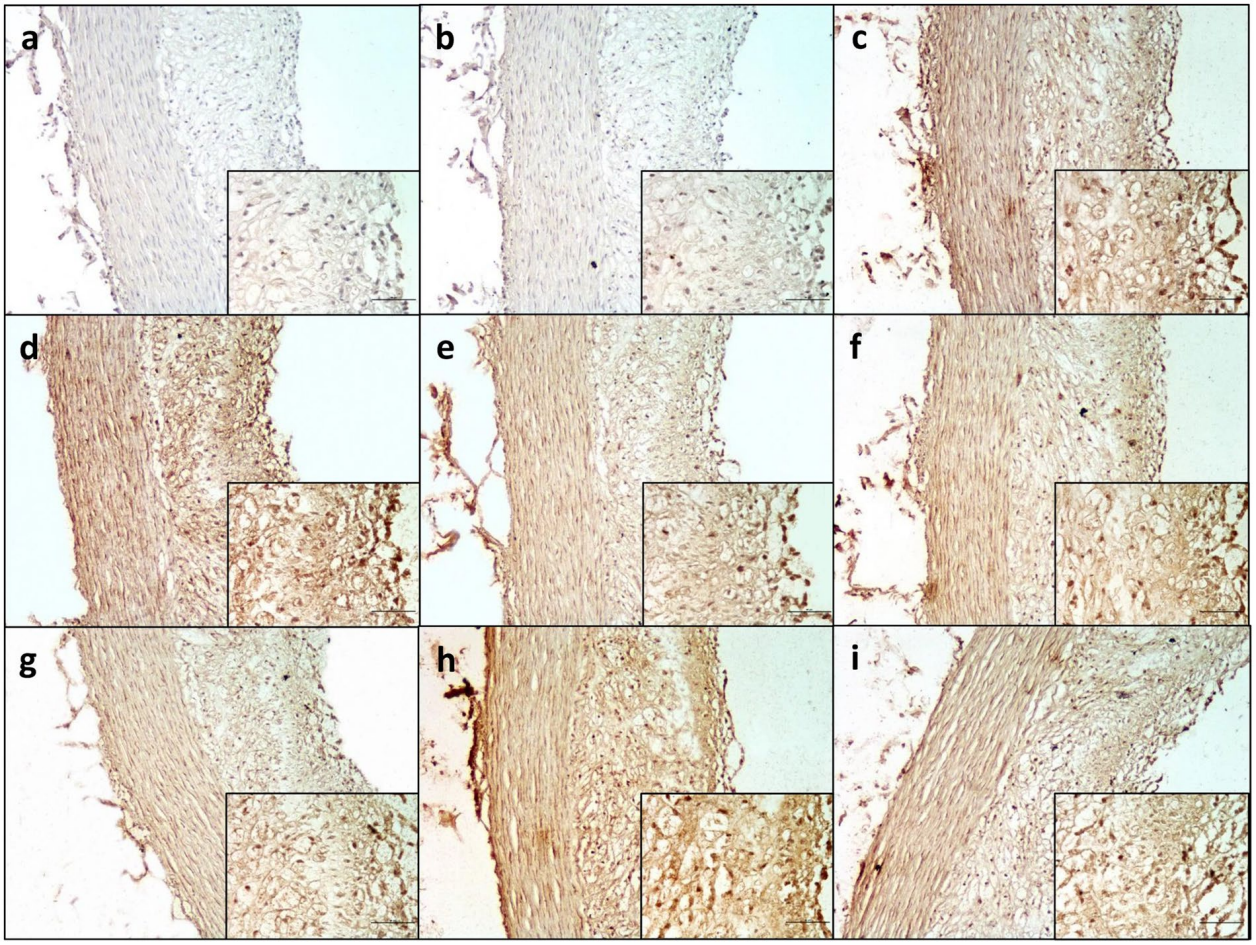
Immunodetection of scFv-phages on sections of vulnerable atherosclerotic lesions from atherosclerotic NZW rabbits by immunohistochemical assays. Selected scFv-phages were incubated with atherosclerotic rabbit tissue sections. (**a**) Secondary antibody alone, (**b**) A2PL20, (**c**) B6PL20, (**d**) B7PL20, (**e**) C3PL20, (**f**) C9PL20, (**g**) C10PL20, (**h**) D3PL20, (**i**) E3PL20. After addition of secondary anti-pVIII protein antibody, sections were revealed with DAB substrate kit reagent. The presence of the antigen recognized by scFv-phages was indicated by a yellow-brown staining. No staining was observed in the presence of secondary antibody only. Scale bars represent 50 µm. A 10X magnification is presented for each scFv-phage. The picture on the right down corner corresponds to a magnification of 40X. Nuclei were counterstained with hematoxylin.

The IHC experiments conducted on eight representative clones with atherosclerotic NZW rabbit tissue sections are shown in Fig. 7. Hyperphages were used as a control at a concentration of 5. 10^9^ phages/mL, determined by ELISA for the eight clones tested (Supplementary Methods S3). B6PL20, C3PL20, C9PL20, C10PL20 and E3PL20 clones (Fig. 6c,e–g,i) bound preferentially the sub-endothelial layer whereas B7PL20 and D3PL20 (Fig. 6d,h) showed a broader reactivity within the intima. We noticed that B7PL20 and D3PL20 reacted with intimal proteins with the same intensity despite a very different expression level of scFv-pIII, underlying the fact that scFv expression is not strikingly correlated to the level of binding. Thus, we show here that even poorly displayed scFv can give rise to good reactivity in IHC experiments, depending on either the representativeness of the target or the affinity of the scFv. Whatever, the IHC experiments corroborate the results obtained with the high-throughput flow cytometry assay. Considering all of our experiments (PL19 to PL27), IHC analyses revealed that 60% of clones, selected by flow cytometry, were able to recognize over-represented proteins within sections of atheroma plaque. The 40% of clones that did not recognize their target in IHC were withdrawn from further studies. This fail in antigen recognition may be more assigned to modification of epitopes occurring after treatment of tissues with paraformaldehyde fixation, and/or high temperatures treatment of the tissue sections than to false positive results in flow cytometry screening. On the contrary, we have experienced some clones initially found negative during the flow cytometry screening step showing in IHC assays tiny reactivity on atheromatous sections. We were initially aware of this pitfall due to limitations in the extraction process, essentially caused by the loss of some proteins and the degradation of some others. Flow cytometry experiment using protein extracts is not a conformational screening strategy, meaning that some scFv could be missed by this approach. However, this strategy has the benefit to offer a higher throughput capacity compared to IHC. Altogether, IHC certifies the reactivity of these pre-screened clones on sections of atheroma and acknowledges the robustness of the flow cytometry screening.

## Discussion

Acute cardiovascular events, due to rupture or erosion of an atherosclerotic plaque, represent the major cause of morbidity and mortality in patients. Growing evidence suggests that plaque vulnerability is directly linked to its molecular and cellular composition, which now places new demands on molecular imaging. It is expected from this rapidly evolving field an accurate assessment of molecular, cellular and compositional components of atherosclerotic lesions to efficiently diagnose high-risk patients. This imaging modality requires highly sensitive and specific probes made of an affinity ligand for targeting and a signal detection compound.

Magnetic resonance imaging (MRI), a non-invasive modality, has a great potential for clinical application considering multiple advantages such as its high resolution and the absence of ionization^22^. Thanks to the knowledge on some biomarkers overexpressed in atherogenesis, researchers developed functionalized contrast agents dedicated to magnetic resonance imaging to target αvβ3 integrin overexpressed in angiogenesis^23^, fibrin involved in thrombosis^24^, annexin V expressed on apoptotic macrophages^25^, oxLDL^26^ and scavenger receptor from foam cells^27^ or P-selectin on activated platelets and endothelial cells^28^. These MRI studies were mainly conducted with nanoparticles coupled to peptides or commercial and home-made murine and rat antibodies.

Because of two major inconveniences, which are the immunogenicity of rodent Abs and the lower affinity of peptides for their targets, the development of human antibodies to gain affinity and decrease immunogenicity is the aim of this study. In the context of atherosclerosis, designing human antibodies as affinity ligands for targeting is very new, except sparse studies of Fab fragments raised against well-defined antigens such as oxLDL^29^. Here another novelty which we consider important to emphasize is the selection of these human antibodies by *in vivo* phage display for accessing a large panel of targets in pathophysiological conditions. Because the environment impacts the folding of proteins and their expression, *in vitro* methods often result in the selection of peptides or antibodies no more able to recognize their target when transferred *in vivo* for pre-clinical and clinical studies. Thus, to get around this issue, we implemented *in vivo* phage display selections that may result in an array of recombinant scFv-phage clones with the potential to recognize various and unknown targets. By extending the selection over three rounds, we aimed to force the enrichment of specific antibodies. This strategy led to a strong increase in scFv-phage titers after the third round.

Because the selection of HuAbs has been made *in vivo*, a crucial step remains the screening for individual scFv-phage clones against unknown proteins. A big challenge was the set-up of a high-throughput screening with low quantities of biological material meanwhile making available a high surface density of different target antigens. The fact that the antigen is unknown limits the use of classic ELISA assays where purified antigens are usually coated on plates at quite high concentrations. Here, we demonstrate that it is highly feasible to screen around thousands of scFv-phage clones by the design of a suitable high-throughput method using flow cytometry and atheromatous proteins coated on beads at low concentration. The clear objective of this assay was to rapidly screen thousands of individual clones among the scFv-phages recovered from the third round of *in vivo* selection. This first flow cytometry screening step was successfully achieved as attested by the selection of more than two hundred of scFv-phage hits, which presented substantiated binding for the three batches of protein-coated beads.

The following steps were conducted to evaluate the germline and juntional diversity of scFv clones by IMGT/V-QUEST bioinformatic system analyzes and to validate their bioreactivity by IHC assays.

Out of two hundred and nine clones selected by flow cytometry, we retrieved 142 clones with complete and in frame VH and VL germline genes after sequencing of the whole scFv fragment. Moreover, analysis by IMGT/V-QUEST has highlighted a high diversity of germline genes and CDR3 junctions, pointing out a potential high diversity in targeting biomarkers. IHC experiments with selected scFv-phage clones have been included (1) to prove the robustness of flow cytometry screening with the reactivity of 60% of selected clones confirmed *ex vivo* on NZW rabbit atheroma sections and (2) to provide visual localisation of these pre-screened clones within atheroma.

Moreover, in this work, proteomic analyses have highlighted hundreds of proteins overexpressed in the context of atherogenesis, validating the atheromatous model used here to screen human antibodies and defining potential new biomarkers. The Pathway Studio analysis has identified a lot of proteins over-represented in the atherosclerosis context and well-known in the literature such as apolipoproteins E, B and A1, integrins, scavenger receptors. On the other side, the analysis has highlighted the presence of proteins, not well assigned in the literature to atherogenesis, which will be interesting to test against selected antibodies. The next challenge will be to identify among the panel of atheromatous proteins in the extract, the targeted molecules, making use of selected scFv produced as scFv-Fc fragments at large-scale.

These high-scale produced human scFv-Fc antibodies could thus help to survey the molecular and cellular components of atheroma plaques, increasing our understanding of different molecular pathways (as seen by proteomics analyses) leading to the development of atheroma.

In future studies, the human scFv-Fc antibodies will serve for *in vivo* experiments on atherosclerotic ApoE^−/−^ mice as suitable moieties after grafting on nanoparticles for (i) a more accurate diagnosis of atherosclerosis dedicated to MRI imaging and (ii) the development of therapeutic strategies. It is to be emphasized that human antibodies which have been selected in a relevant animal model of atherosclerosis, would allow direct transfer from preclinical to clinical studies. The human antibodies that will be used in pre-clinical studies will be those able to target the corresponding human proteins on human atheroma sections.

The strategy of selection by *in vivo* phage display coupled to high-throughput screening by highly sensitive flow cytometry which has the capability to recognize proteins underrepresented in the extract could be applied to many other studies in cancer and auto-immune diseases for identification of target/ligand pairs.

## Methods

### Complex plaque formation in the NZW rabbit animal model

All animal experiments were performed in accordance with the Guide for the Care and Use of Laboratory Animals (NIH Publication No. 85-23, revised 1996) and were approved by the ethic committee of Bordeaux (CEEA50). The NZW rabbit model was fed with a high cholesterol diet as previously described^13^ and two surgeries were performed, two months apart. The first consisted in a desendothelialization from the thoracic until the abdominal aorta and the second in an angioplasty carried out with a balloon through a femoral arteriotomy to the descendent thoracic aorta under radioscopic guidance.

### *In vivo* phage display selection in the atherosclerotic NZW rabbit model

#### ScFv-phage library preparation

The fully human recombinant fragment scFv antibody library described in patent WO2007137616 was used for *in vivo* phage display selection. For phage rescue, the phage library was infected with M13-KO7 helper phage (Invitrogen, France), followed by precipitation of phages with PEG/NaCl. After centrifugation at 11,000 g for 45 min at 4 °C, the pellet containing scFv-phage particles was resuspended and filtered in a final volume of 500 µL sterile cold PBS.

#### *In vivo* phage-display

Three rounds of biopanning were performed by phage display in atherosclerotic NZW rabbits. The full scheme of the procedure is given in Fig. 2. The first selection was carried out using a continuous flow (150 µL/min) of 2.4 × 10^12^ colony-forming units (cfu) of scFv-phages injected into the NZW rabbit marginal ear vein for 30 min. After 1 h circulation, the animal was sacrificed and perfused via the heart with 120 mL of PBS to ensure phage clearance from the blood. The aorta was recovered from the aortic arch to the iliac bifurcations and divided into E1 (aortic arch and thoracic area) and E2 (abraded renal and abdominal areas) sections as shown in Fig. 2. Rounds 2 and 3 were conducted following the same procedure, excepted that the quantity of scFv-phages was lowered to 4.8 × 10^11^ and 3.9 × 10^11^ cfu respectively.

To access not only the scFv-phages binding to the endothelial cell surface but also those migrating into the intima and those internalized into cells invading the lesions, we respectively recovered F1, F2 and F3 fractions from aortic tissue (Fig. 2).

Details concerning the recovery of the different fractions are given in the supplementary data section (Supplementary Methods S4).

ScFv-phage fractions F1, F2 and F3 were separately rescued by infecting XL1-blue *E*. *coli* (Stratagene, France) (OD = 0.5) and the cells were pelleted and plated on ampicillin (100 µg/mL) and glucose (2% (w/v)) containing 2xTY agar (2TYGA) and incubated 16 h at 30 °C. Spreading on 145 mm Petri dishes has allowed constituting the glycerol stock and limiting dilutions on 80 mm dishes has allowed evaluating the enrichment of selection. After the third round of biopanning, plated clones were individually picked into deep well 96 masterblocks filled with 2TYGA selective medium (Greiner Bio one, France) for flow cytometry screening and further analyses.

### Protein extraction and mass spectrometry study

#### Protein extraction

The protein extraction was performed as previously described^7^. Briefly, BIAHR and H proteins were solubilized with a commercially available T-PER lysis buffer (Thermo Fisher Scientific, France) complemented with a protease inhibitors cocktail (Thermo Fischer scientific, France). Solubilization was performed using a Polytron TP-20 Homogenizer 8 (Kinematica, Lucerne, Switzerland). After two centrifugations, at 13,000 g for 45 min at 4 °C to discard insoluble material from the supernatant, the protein concentration of every soluble extract was determined using a Bradford assay kit according to the manufacturer’s instructions (Thermo Fisher Scientific, France).

#### Mass spectrometry of protein extract

The diversity of proteins in the aorta extracts was assessed by mass spectrometry at the Centre de génomique fonctionelle of Bordeaux.

nLC-MS/MS analysis and database search: Ten µg of proteins were loaded in Laemlli buffer onto SDS-PAGE under reducing condition using 10% gels. Separation was stopped once proteins have entered resolving gel. Colloidal blue stained bands were cut out from the gel and treated as described elsewhere^30^. Briefly, gel pieces were destained and digested with trypsin overnight at 37 °C. Peptides were extracted and injected on a Ultimate 3000 nanoLC system (Dionex, Amsterdam, The Netherlands) coupled to a Electrospray Q-Exactive mass spectrometer (Thermo Fisher Scientific, San Jose, CA). Mass spectrometry data were acquired in a data-dependent mode alternating a MS scan survey on *m/z* 300–2000 with HCD (High Collision Dissociation) MS/MS spectra on top 15 ions. Data were searched by SEQUEST through Proteome Discoverer 1.4 (Thermo Fisher Scientific Inc, France) against a subset of the 2014.05 version of UniProt database restricted to *Homo sapiens* Reference Proteome Set (68,358 entries). Tolerances were set to 10 ppm and 0.02 Da for precursors and fragments respectively. Only b- and y-ions were considered for mass calculation. Oxidation of methionines and deamidation of asparagines and glutamines were considered as variable modifications and carbamidomethylation of cysteines as fixed modification. Two missed trypsin cleavages were allowed. Peptides were validated at a False Positive Rate of 1% using Percolator^5^. Label-free quantitative data analysis was performed using Progenesis LC-MS 4.1 software (Nonlinear Dynamics Ltd, U.K). Importantly, normalization was based on median ratio, and only non-conflicting features and unique peptides were considered for calculation. Quantitative data were considered for proteins quantified by a minimum of 2 peptides.

Functional analysis: Protein profiles of the aortic arch and thoracic aorta from healthy and balloon-injured aortas of hypercholesterolemic NZW rabbit were compared. Ratios of normalized protein abundance between BIAHR and H extracts were calculated and imported in Pathway Studio 11.3 for functional analysis and biological meaning. GSEA-based (SNEA) and Fisher’s based (FSNE) cell-process subnetwork enrichment analysis tests were performed using default settings on the entire ratio dataset and the list of top 100 over-represented proteins respectively. To help with biological interpretation, top 100 cell processes found enriched in each analysis test were combined, further categorized, and checked for the existence of functional association with the atherosclerosis entity in Pathway Studio. The sentences of all relationships supported by less than three references were closely reviewed, and only highly confident links were reported.

### ScFv-phage preparation

Individual recombinant XL1-blue colonies from the third biopanning were grown overnight at 30 °C and shaken at 280 rpm (Newbrunswick, Edison, USA), in 96-deep well plates (masterblock) containing 500 µL of 2TYGA medium supplemented with 10 µg/mL of tetracyclin (Tet). Twenty-five microliters of this previous culture were used to inoculate 500 µL of 2TYGAT medium for the scFv-phage production. After 3 hours of bacteria growth, phage production was induced by adding 25 µL of 2TY medium containing 3 × 10^8^ hyperphage (Progen, Germany). After 1 h of infection at 37 °C, bacteria were then pelleted and supernatants discarded. Infected bacteria were subsequently resuspended in 500 µL of 2TYA medium supplemented with 40 µg/mL kanamycin (Kan) for a last growth overnight at 26 °C, 260 rpm. Bacteria were finally spun down at 10,000 g, 10 minutes and supernatants were immediately used for flow cytometry analyses.

### Phage quantification by ELISA

Wells of flat bottom corning costar 96-well EIA/RIA plates (Fischer Scientifc, France) were coated overnight at 4 °C with mouse anti-M13 (specific to pVIII protein) monoclonal antibody (GE healthcare, U.K) diluted at 5 μg/mL in carbonate buffer (15 mM carbonate, 35 mM bicarbonate/pH 9.6). Wells were then blocked with 5% MPBS (milk PBS). Dilutions of scFv-phage supernatants (1:10 and 1:100) and a range of hyperphage (Progen, Germany) from 5.10^8^ to 10^11^ cfu/mL were added to wells in duplicates and incubated for two hours at RT. After washing, HRP-conjugated anti-pVIII protein monoclonal antibody (GE healthcare), diluted 1:5,000 in 2% MPBS, was added for 1 hour at RT. Each step was followed by extensive washing in PBS Tween-20 0.5% buffer (TPBS). The final washing was carried out with PSB alone and color was developed with OPD system for ELISA (Sigma Aldrich, France). Color absorbance was immediately read at 405 nm in a plate reader (Chameleon, Thermofisher, France).

### Flow cytometry analyses

#### Coupling of proteins to magnetic beads

Fifty micrograms of BIAHR proteins were covalently coupled to 300 nm carboxyl-adembeads according to the manufacturer instructions (Ademtech, France). Three batches of coated protein beads per sample were used for reproducibility. The coupling rate of BIAHR proteins on carboxyl-adembeads was analysed on SDS-PAGE under reducing condition using 4–15% gels (miniprotean TGX gel, Biorad, France). The rate of uncoated fractions which migrated in the gel was determined by using the ImageJ software.

#### ScFv-phage screening by flow cytometry

Specificity of scFv-phages to BIAHR proteins was determined by flow cytometry in 96-well plates (Becton Dickinson, France). Forty microliters of BIAHR proteins coated on beads were added at 5 µg/mL to 100 µL of phage particles (range of hyperphage from 10^8^ to 10^10^ cfu/mL) or individual scFv-phage and were incubated overnight at 4 °C. Different experimental conditions (A, B, C and D) were first tested to evaluate the rate of non-specific binding of hyperphage particles on coated protein-beads (supplementary Fig.S3). These conditions, summarized in Table 1, differ in the number of centrifugations (10 min at 8,000 g) and washes performed before or after addition of a murine anti-pVIII primary antibody (Abcam, France) at 1 µg/mL for one hour at RT, 150 rpm. The Alexa 488 labeled anti-mouse antibody (Life technologies, France) was used at 3.75 µg/mL for final phage detection. To evaluate the coupling efficiency and quality of proteins on beads, murine AP-2 antibody (courtesy of Dr Alan Nurden) (anti-αIIbβ3 antibody) and rabbit anti-carbonic anhydrase II (CAII) antibody (Abcam, France) were incubated in the same conditions as above with the difference that secondary Alexa 488 labeled antibodies were used at 5 µg/mL (goat anti-mouse) or 3 µg/mL (goat anti-rabbit) for the detection of AP2 or CAII respectively. Samples were analysed on a BD FACS Canto I device (Becton Dickinson, France) using software Diva. All washes and dilutions were performed in PBS. Fluorescence geomean was statistically calculated on the basis of 10,000 fluorescent events.

## Electronic supplementary material

Supplementary information 1

Supplementary information 2
