# Supplementary material for: An innovative flow cytometry method to screen human scFv-phages selected by in vivo phage-display in an animal model of atherosclerosis
Source: Sci Rep. 2018 Oct 9;8:15016. doi: 10.1038/s41598-018-33382-2 (PMC6177473; doi:10.1038/s41598-018-33382-2)
Supplement: Supplementary file 1 — Supplementary information 1 [file 41598_2018_33382_MOESM1_ESM.pdf]

An innovative flow cytometry method to screen human scFv-phages selected by *in vivo* phage-display in an animal model of atherosclerosis

Audrey HEMADOU[1], Jeanny LAROCHE-TRAINEAU[†1], Ségolène ANTOINE[†1], Philippe MONDON[2], Alexandre FONTAYNE[2], Yannick LE PRIOL[3], Stéphane CLAVEROL[4], Stéphane SANCHEZ[1], Martine CERUTTI[5], Florence OTTONES[1], Gisèle CLOFENT-SANCHEZ[†1] and Marie-Josée JACOBIN-VALAT[†*1]

[†]Equivalent position of authors

[1] CRMSB, UMR5536 CNRS, INSB, Bordeaux, 33076, France

[2] LFB Biotechnologies, department of biotherapeutic engineering Lille, 59000, France

[3] Elsevier Masson SAS, Elsevier RD solutions, Issy les Moulineaux, 92130, France

[4] CGFB, Proteome pole, Bordeaux, 33076, France

[5] UPS 3044, CNRS, Saint-Christol-Lès-Alès, France

| Gene Set Seed | Category | TOP 100 SNEA | SNEA p-value | Measured Entities | TOP 100 FSNE | FSNE p-value | Overlapping Entities | Connected to athero-sclerosis | Reported in Figure 3a |
|---|---|---|---|---|---|---|---|---|---|
| Hemolysis | Vascular | Y | 7.6E-06 | PTPRC;ENO2;ITGB2;ALB;CLU;BAX;RAC2;PLG;A2M;C4A;LTF;TF;APOA1;APOB;HPX;HP;CP;XDH;HPRT1;VTN;CAT;APP;TXN;FN1;G6PD;F2;AQP1;LDHA;GPI;TPI1;CALR;HBB;ANXA5;RPL19;CAV1;GSR;HBA1;RAC1;ITPA;HSPA9;PRDX2;PHB;HSP90B1;CAPN1;EEF1A1;C3;ROCK1;HADHA;ACP1;PRELP;DCN | Y | 2E-10 | PTPRC;ENO2;ITGB2;BAX;APOB;LTF;APOA1;RAC2;PLG;HP;ALB;C4A;XDH;CP;TF;HPRT1;A2M;HPX;CLU | N | - |
| Agglutination | Vascular | Y | 2.8E-05 | ACTN1;ALB;CLU;PLG;A2M;LTF;TF;LGALS3;FLOT2;HP;CP;APP;FN1;F2;FABP9;HSPA8;CD36;HSPD1 | Y | 4E-10 | LGALS3;CP;TF;LTF;A2M;FLOT2;CLU;PLG;HP;ALB;ACTN1 | N | - |
| Liver uptake | Miscelleneaous | Y | 7.3E-05 | PLIN2;ALB;A2M;LTF;TF;LGALS3;APOA1;APOB;HPX;APOE;HP;CP;XDH;CAT;APP;FN1;P4HB;LRP1;CD36;AOC3;C3;ITGB5;DCN | Y | 6E-12 | LGALS3;APOB;LTF;APOA1;HP;ALB;XDH;CP;TF;A2M;HPX;APOE;PLIN2 | N | - |
| Opsinization | Lipid-related | Y | 0.00066 | ITGB2;ALB;PLG;C4A;LGALS3;APOB;MFGE8;APOE;VTN;APP;FN1;ANXA2;CALR;ITGA5;C3;VIM | Y | 6E-09 | C4A;LGALS3;ITGB2;APOB;APOE;MFGE8;PLG;ALB | N | - |
| Macrophage function | Immunology | Y | 0.0008 | PTPRC;FABP5;ITGB2;ACTN1;ALB;CLU;RAC2;CAPG;PLG;A2M;LTF;C5;TF;LGALS3;APOA1;CORO1A;LGALS3BP;APOE;HP;UFM1;TNFRSF6B;CAT;PPP5C;APP;FN1;F2;SERPINF1;ANXA5;MAPK3;CAV1;SET;STAT1;RAC1;ANXA1;PPAP2A;RHOA;CD9;CDC42;LRP1;CD36;GRB2;HSPD1;EPRS;PHB;IQGAP1;HSP90B1;CYBB;MAPK1;HMGB1;SLC25A1;ROCK1;STAT3;GSK3B;PTPN11;GPX1;MFAP4;TGM2;PALLD;PTK2 | Y | 3.2E-13 | PTPRC;LGALS3;ITGB2;LGALS3BP;C5;LTF;RAC2;APOA1;PLG;HP;ACTN1;ALB;TF;A2M;APOE;FABP5;CLU;CAPG;CORO1A | Y | Y |

| | | | | | | | | | |
|---|---|---|---|---|---|---|---|---|---|
| Antigen binding | Immunology | Y | 0.00086 | ALB;CLU;BAX;HTRA1;PLG;A2M;LTF;CTSH;TF;APOB;ITGB3;APOE;MMP2;APP;FN1;G6PD;HLA-DQB1;ANXA4;F2;ANXA2;FOLH1;EEA1;ANXA1;RHOA;DES;RPL14;MAPK1;HMGB1;PURA;ACSL1;GALNT2;TLN1;NCAM1;TROVE2;TGM2;PTK2 | Y | 5E-09 | HTRA1;BAX;APOB;LTF;PLG;ALB;CTSH;TF;APOE;A2M;CLU;ITGB3 | N | - |
| Complement activation | Immunology | Y | 0.0013 | ITGB2;ALB;CLU;COMP;HTRA1;PLG;HRG;A2M;C4A;LTF;C5;TF;APOA1;C6;APOE;HP;XDH;VTN;ITIH2;MMP2;APP;TXN;FN1;F2;ENO1;GPI;ANXA2;TPI1;CALR;NID1;P4HB;SERPINB1;GAPDH;BGN;HSPD1;HSP90AB1;PHB;SOD2;HMGB1;SERPINA3;EFEMP1;KRT1;C3;VIM;PRSS1;FMOD;PRELP;DCN;HAPLN1 | Y | 1.9E-14 | C6;HTRA1;ITGB2;LTF;COMP;C5;HRG;APOA1;PLG;HP;ALB;C4A;XDH;TF;A2M;APOE;CLU | Y | - |
| Neutrophil phagocytosis | Immunology | Y | 0.00153 | PTPRC;ITGB2;ALB;LCP1;LTF;LGALS3;CORO1A;HPX;MFGE8;HP;CP;FN1;ANXA5;MAPK3;ANXA1;LRP1;CD36;GAPDH;MAPK1;HMGB1 | Y | 7E-13 | PTPRC;LGALS3;CP;ITGB2;LTF;HPX;LCP1;MFGE8;HP;CORO1A;ALB | N | - |
| Cell recognition | Immunology | Y | 0.00161 | PTPRC;ITGB2;ALB;PLG;LTF;C5;TF;LGALS3;APOA1;APOB;ITGB3;MFGE8;APOE;FN1;HLA-DQB1;HLA-B;PKM;ANXA2;CALR;MSN;STAT1;ANXA1;CDH13;VCAN;CTNNA1;RPL11;LRP1;CD36;PGM3;ACTR2;DES;PRDX5;ACTR3;STAT3;PTPN11;ITGB1;PCDH7;NCAM1;PRAF2;TGM2;MARCKS;NRP1 | Y | 1E-11 | PTPRC;LGALS3;ITGB2;APOB;C5;LTF;MFGE8;APOA1;PLG;ALB;TF;APOE;ITGB3 | N | - |
| Phagocyte activity | Immunology | Y | 0.0025 | PTPRC;ITGB2;ALB;RAC2;GUSB;CAPG;GC;PLG;HRG;A2M;LTF;LGALS3;APOA1;ITGB3;MFGE8;APOE;HP;VTN;TNFRSF6B;CAT;APP;FN1;ANXA3;F2;CLIC1;CALR;ANXA5;NID1;MAPK3;RAC1;SNAP23;EIF5A;ANXA1;RHOA;ITGAV;RCN1;CDC42;LRP1;CD36;PRKCD;PPP2R4;HSPD1;PSMD4;PRDX5;CYBB;MAPK1;PPIA;HMGB1;C3;ROCK1;STAT3;VIM;ROCK2;DAG1;DPYSL3;ARF6;RAP1A;INPP4A;MYLK;PTK2;DYSF;ELN | Y | 6E-11 | PTPRC;LGALS3;ITGB2;LTF;HRG;RAC2;APOA1;MFGE8;PLG;HP;ALB;GUSB;A2M;APOE;ITGB3;CAPG;GC | N | - |
| Macrophage chemotaxis | Immunology | Y | 0.0052 | ITGB2;CLU;GC;A2M;C4A;LTF;LGALS3;APOA1;S100A4;APP;TXN;ANXA3;F2;MAPK3;RAC1;FBN1;RPS19;CYBB;MAPK1;HMGB1;ROCK1;ELN | Y | 9.3E-09 | C4A;LGALS3;ITGB2;S100A4;A2M;LTF;CLU;APOA1;GC | Y | - |
| Lipid oxidation | Lipid-related | Y | 0.0057 | ENO2;ALB;CLU;BAX;PON3;ACOX1;LTF;TF;APOA1;APOB;APOE;HP;CP;CAT;APP;DPP4;DLD;GSR;HSD17B10;AIFM1;PRDX2;ATIC;PRDX3;SOD2;ARF1;STAT3;GPD1;STK25;GNAS;GPX1;ACADM | Y | 1.7E-09 | ENO2;BAX;PON3;APOB;LTF;APOA1;HP;ALB;CP;TF;APOE;CLU;ACOX1 | Y | Y |
| Lactation | Miscelleneaous | Y | 0.00631 | PLIN2;ALB;CLU;BAX;PLG;IGFBP7;LTF;TF;APOA1;CTSD;MFGE8;APOE;HP;CP;DAB2;XDH;HPRT1;FN1;G6PD;COX2;AQP1;ACLY;ALAD;ANXA5;HK1;LNPEP;IDH1;CAV1;RAC1;PGM1;HSPA8;YWHAG;CDC42;CD36;GAPDH;ITGA5;PRPS1;UGP2;GLS;SOD2;MAPK1;SERPINA3;SELENBP1;STAT3;ACTA2;PTPN11;DAG1;ITGB1;ACTB;ATP2B4;NCAM1;MYLK;ATP2A3;FES;PLIN1;PTK2;MYH11 | Y | 5E-12 | DAB2;BAX;LTF;APOA1;MFGE8;PLG;HP;ALB;CTSD;XDH;CP;IGFBP7;TF;HPRT1;APOE;CLU;PLIN2 | N | - |

| | | | | | | | | | |
|---|---|---|---|---|---|---|---|---|---|
| Pregnancy | Miscelleneaous | Y | 0.0064 | PTPRC;ITGB2;PLIN2;ALB;CLU;BAX;SDCBP;GUSB;HTRA1;HNMT;GC;PLG;HRG;A2M;IGFBP7;FTH1;ACOX1;LTF;CTSH;TF;LGALS3;FLII;APOA1;HEXA;APOB;CTSD;ITGB3;HPX;GLB1;S100A4;MFGE8;APOE;HP;COL1A1;CP;DAB2;XDH;HPRT1;TNFRSF6B;CAT;HSPE1;MMP2;WARS;TXN;S100A11;FN1;G6PD;ANXA4;NCL;F2;AQP1;ACLY;LDHA;SOD3;LAMB1;ENO1;GPI;FABP4;DPP4;PRDX1;GSN;PTGES3;CALR;ANXA5;SERPINF1;HBB;EPHX1;RALA;LNPEP;MAPK3;AKR1B1;IDH1;CAV1;SET;GSR;HBA1;STAT1;CACYBP;ELAVL1;CKM;RAC1;TPT1;GPX3;MTHFD1;PPAP2A;RHOA;NDRG2;GSTP1;HSPA8;CD9;PGRMC1;LRP1;CD36;ALDOA;IGF2R;DES;PRKCD;GAPDH;PHB2;PITPNA;BGN;CRYAB;PARK7;HSPD1;ITGA5;LDHB;PRKAR2A;PDHA1;PRPS1;NPEPPS;HNRNPA1;UGP2;NT5E;GNAI3;PTGIS;MAPK1;PPIA;HSPB6;ITGA1;SOD2;ARF1;NDUFA13;AKR1C1;DCXR;ANXA6;GNB2L1;FH;HSPA2;PAFAH1B1;HSPG2;RELA;STAT3;CYB5R3;TMED2;ROCK1;VCL;HADHA;VIM;PDE5A;RPSA;ROCK2;DAG1;ACTB;GSK3B;ACTA2;ITGB1;GNAS;GNAQ;HRNR;COL4A1;ATP2B4;EPHX2;ARF6;GDI2;CBR1;KRT16;SFPQ;HSPB1;ATP2A2;COL4A2;GPX1;DCN;MPST;NCAM1;ILK;PDE3A;TGM2;MYLK;CALD1;SVIL;SERPINH1;PLIN1;PTK2;FBLN5;NRP1;ELN;PXDN | Y | 5.2E-22 | HTRA1;APOB;LTF;HNMT;HRG;MFGE8;SDCBP;PLG;GLB1;HP;ALB;CTSD;COL1A1;FLII;XDH;CP;IGFBP7;TF;APOE;CLU;ACOX1;PLIN2;DAB2;PTPRC;LGALS3;ITGB2;HEXA;BAX;APOA1;GUSB;CTSH;HPRT1;S100A4;A2M;HPX;ITGB3;FTH1;GC | Y | - |
| Macrophage phenotype | Immunology | Y | 0.0089 | FABP5;ACTN1;COMP;RAC2;A2M;LGALS3;APOA1;HPX;MFGE8;APOE;HP;CES1;CAT;APP;TXN;STAT1;RAC1;CD36;CRYAB;HSPD1;CYBB;MAPK1;HMGB1;ACSL1;STAT3;GSK3B;PTK2 | Y | 4.9E-10 | LGALS3;COMP;HPX;APOE;A2M;FABP5;MFGE8;APOA1;RAC2;HP;ACTN1 | Y | Y |
| Neutrophil activation | Immunology | Y | 0.0103 | PTPRC;ITGB2;ACTN1;ALB;BAX;RAC2;LCP1;A2M;C4A;LTF;LGALS3;APOA1;LGALS3BP;APOB;APOE;HP;XDH;CAT;APP;TXN;F2;CALR;MAPK3;CAV1;RAC1;DDOST;RPL22;ANXA1;YARS;VCAN;CDC42;SERPINB1;CTSC;CYBB;MAPK1;HMGB1;SERPINA3;RAN;ITGAL;C3;ROCK1;STAT3;AOC3;GSK3B;PTPN11;TLN1;RAP1A;MARCKS;CRKL | Y | 1.0E-13 | PTPRC;LGALS3;LGALS3BP;ITGB2;BAX;APOB;C5;LTF;LCP1;APOA1;RAC2;HP;ALB;ACTN1;C4A;XDH;A2M;APOE | Y | Y |
| Engraftment | Miscelleneaous | Y | 0.01368 | PTPRC;ITGB2;ALB;COMP;RAC2;LCP1;LTF;TF;LGALS3;ITGB3;FLOT2;APOE;DAB2;VTN;CAT;MMP2;TXN;FN1;HLA-DQB1;F2;DPP4;ANXA2;SERPINF1;CAV1;STAT1;RAC1;GSTP1;RHOA;SBDS;CDC42;PAK2;RECK;ITGA5;CYBB;SOD2;HSP90B1;HMGB1;ITGAL;STAT3;GSK3B;PTPN11;ITGB1;GNAS;IDH2;RAP1A;HSPB1;SPARC;LASP1 | Y | 3E-09 | PTPRC;DAB2;LGALS3;ITGB2;COMP;LTF;FLOT2;LCP1;RAC2;ALB;TF;APOE;ITGB3 | N | - |

| | | | | | | | | | |
|---|---|---|---|---|---|---|---|---|---|
| Cell interaction | Immunology; Vascular | Y | 0.0152 | FABP7;PTPRC;ITGB2;ACTN1;ALB;CLU;BAX;HTRA1;PLG;HRG;A2M;LTF;TF;LGALS3;FLII;APOA1;SCARB2;LGALS3BP;APOB;ITGB3;SERPINE2;MFGE8;APOE;HP;DAB2;VTN;CAT;MMP2;APP;TXN;FN1;HLA-B;NCL;F2;AQP1;SOD3;FABP4;DPP4;S100A10;ANXA2;CALR;ANXA5;SERPINF1;NID1;MAPK3;CAV1;STAT1;TGFBI;MSN;RAC1;TPT1;ANXA1;PPIB;CDH13;RHOA;ITGAV;CTNNA1;P4HB;VCAN;CD9;CDC42;LRP1;CD36;FSCN1;PRKCD;GAPDH;BGN;CRYAB;EPRS;PAK2;AIP;HSPD1;RECK;ITGA5;FAP;NT5E;MAPK1;PPIA;HMGB1;IQGAP1;CYBB;SOD2;VASP;HYOU1;PPP2R1A;ANXA6;CRK;ACSL1;HSPG2;EEF1A1;ITGAL;STAT3;AOC3;ROCK1;VCL;VIM;DAG1;TLN1;GSK3B;ITGB1;RRAS;COL4A1;ARF6;FUBP1;CSPG4;ITGB5;RAP1A;JUP;MFAP4;COL4A2;DCN;NCAM1;PPP1R12A;SPARC;ILK;CTTN;PPP1R14A;TGM2;RPL12;ENAH;MRVI1;SYNM;MARCKS;PTK2;FBLN5;DYSF;CRKL;NRP1;ELN | Y | 1.4E-15 | HTRA1;APOB;LTF;HRG;MFGE8;PLG;HP;ALB;COL1A1;FLII;TF;APOE;FABP7;CLU;PTPRC;DAB2;LGALS3;LGALS3BP;ITGB2;BAX;APOA1;SERPINE2;ACTN1;SCARB2;A2M;ITGB3 | Y | - |
| Ossification | Vascular | Y | 0.0157 | ITGB2;ALB;CLEC3B;COMP;GUSB;HTRA1;GC;PLG;IGFBP7;FTH1;LTF;TF;LGALS3;APOA1;CTHRC1;ITGB3;S100A4;APOE;FBLN1;COL1A1;CP;XDH;VTN;MMP2;TXN;FN1;FLNB;F2;FKBP1A;GLG1;ANXA2;ALAD;CALR;ANXA5;SERPINF1;MAPK3;PPID;CAV1;STAT1;TGFBI;RAC1;DCLK1;ARL6IP5;YWHAB;COL15A1;RHOA;PGK1;VCAN;CD9;SBDS;CDC42;VPS35;LRP1;HSPA9;PRKCD;FBN1;LMNA;BGN;HSPD1;ITGA5;NT5E;GLS;MAPK1;SOD2;NPNT;PPP3CA;ALDH7A1;DNM2;HSPG2;ASPN;DSTN;STAT3;AOC3;HMGB2;C3;ROCK1;DPYSL3;GSK3B;PRKAR1A;ACTA2;ITGB1;GNAS;PCDH7;FMOD;PRELP;RAP1A;PDLIM7;HSPB1;ATP2A2;TAGLN;DCN;FERMT2;CHKB;NCAM1;SPARC;COL1A2;CNN1;TGM2;OGN;COL6A1;ZYX;HAPLN1;PTK2;NRP1;ELN | Y | 5.2E-11 | CTHRC1;LGALS3;ITGB2;HTRA1;LTF;COMP;RAC2;APOA1;PLG;CLEC3B;ALB;COL1A1;XDH;GUSB;CP;IGFBP7;TF;FBLN1;APOE;S100A4;ITGB3;FTH1;GC | Y | Y |
| Macrophage migration | Immunology | Y | 0.0166 | PTPRC;ITGB2;CLU;RAC2;PLG;A2M;ACOX1;LTF;LGALS3;APOA1;ITGB3;DAB2;VTN;CAT;MMP2;APP;FN1;SPON1;ANXA3;F2;AQP1;SOD3;NQO1;S100A10;ANXA2;SERPINF1;CALR;MAPK3;CAV1;STAT1;TGFBI;RAC1;ANXA1;PPIB;RHOA;CDC42;FSCN1;LRP1;CD36;RAB14;GRB2;BGN;IQGAP1;CYBB;GNAI3;MAPK1;PPIA;HMGB1;VASP;GNB2L1;GNAI2;ITGAL;ROCK1;STAT3;AOC3;HSPB1;TGM2;MARCKS;PTK2;NRP1;ELN | Y | 1.3E-09 | PTPRC;DAB2;LGALS3;ITGB2;LTF;RAC2;APOA1;PLG;A2M;CLU;ITGB3;ACOX1;FTH1 | Y | Y |

| | | | | | | | | | |
|---|---|---|---|---|---|---|---|---|---|
| Adaptive immune response | Immunology | Y | 0.0185 | PTPRC;ITGB2;ALB;CLU;RAC2;LCP1;A2M;ALDH1A2;C4A;LTF;C5;LGALS3;APOA1;CORO1A;C6;LGALS3BP;APOB;MFGE8;APOE;HP;XDH;TNFRSF6B;CAT;HSPE1;APP;MPZ;FN1;HLA-B;F2;SOD3;ENO1;DPP4;CALR;HBB;MAPK3;STAT1;ANXA1;HSPA8;LRP1;CD36;UBE2L3;CTSC;PHB2;BGN;CRYAB;HSPD1;RPS19;HSP90B1;NT5E;CYBB;SOD2;MAPK1;PPIA;HMGB1;NDUFA13;GNAI2;HYOU1;C3;RELA;STAT3;AOC3;GSK3B;CSPG4;DCN;TGM2;LTA4H;PTK2 | Y | 3.1E-13 | PTPRC;LGALS3;C6;ITGB2;LGALS3BP;APOB;LTF;C5;LCP1;APOA1;RAC2;MFGE8;HP;ALB;C4A;XDH;ALDH1A2;APOE;A2M;CLU;CORO1A | Y | Y |
| Macrophage activation | Immunology | Y | 0.0201 | PTPRC;FABP5;ITGB2;ALB;GC;PLG;HRG;A2M;C4A;LTF;TF;FLII;LGALS3;APOA1;CORO1A;APOE;HP;VTN;TNFRSF6B;CAT;APP;HSP90AA1;FN1;PKM;PRDX1;ANXA2;SERPINF1;CALR;ANXA5;MAPK3;CAV1;STAT1;ANXA1;UBE2N;GSTP1;NDRG2;VCAN;CD9;AHCY;CD36;GRB2;CTSC;BGN;HSPD1;EPRS;PARK7;HSP90B1;NT5E;MAPK1;ITGA1;HMGB1;VASP;SETD7;SLC25A1;RELA;STAT3;GSK3B;PTPN11;HNRNPK;RAP1A;TGM2;FES | Y | 6.0E-11 | PTPRC;LGALS3;ITGB2;LTF;HRG;APOA1;PLG;HP;ALB;C4A;FLII;TF;A2M;APOE;FABP5;CORO1A;GC | Y | Y |
| Intestinal absorption | Miscelleneaous | Y | 4.9E-05 | ALB;GUSB;TF;APOA1;APOB;APOE;CP;CES2;CES1;ACAT1;AQP1;ACO1;DPP4;FOLH1;PRMT1;CD36 | N | - | - | N | - |
| Erythrocyte aggregation | Vascular | Y | 9.7E-05 | ALB;CLU;A2M;TF;HP;CP;FN1;F2 | N | - | - | N | - |
| Bacteriostasis | Immunology | Y | 0.00017 | ALB;A2M;LTF;TF;XDH;CAT;TXN | N | - | - | N | - |
| Blood circulation | Vascular | Y | 0.0002 | ALB;PON3;PLG;HRG;TF;APOA1;CTSD;APOE;HP;CP;CAT;MMP2;APP;F2;PKM;GLG1;DPP4;GSTA5;SERPINF1;ANXA5;LNPEP;ANXA1;DES;NDUFA13;HSPG2;C3;KRT5;PDE5A;ILK;KRT14;ELN | N | - | - | Y | - |
| Hemagglutination | Vacular | Y | 0.00022 | ALB;A2M;LTF;TF;LGALS3;FN1;F2 | N | - | - | N | - |
| Macrophage population | Immunology | Y | 0.0007 | PLIN2;TF;LGALS3;APOA1;APOE;CES1;CAT;FN1;ACAT1;DPP4;ANXA5;LRP1;CD36;STAT3;DCN;TGM2;ELN | N | - | - | Y | - |
| Lipoprotein oxidation | Lipid-related | Y | 0.0007 | ALB;APOA1;APOE;CP;CAT;APP | N | - | - | Y | Y |
| Iron absorption | Miscelleneaous | Y | 0.00075 | FTH1;LTF;TF;APOE;CP;XDH;F2;CALR;PRMT1 | N | - | - | N | - |
| Lymphocyte transformation | Immunology | Y | 0.00104 | ALB;A2M;FTH1;TF;HP;FN1;RHOA | N | - | - | N | - |
| Involution | Miscelleneaous | Y | 0.00122 | ALB;BAX;GUSB;HTRA1;PLG;IGFBP7;LTF;CTSD;MFGE8;DAB2;PSAP;MMP2;SSR1;AEBP1;F2;SERPINF1;STAT1;RAC1;RHOA;PRKCD;PHB;MAPK1;CAPN1;STAT3;ITGB1;RAP1A;COL4A2;BIN1;CSRP1;PTK2 | N | - | - | N | - |

| | | | | | | | | | |
|---|---|---|---|---|---|---|---|---|---|
| Lymphocyte apoptosis | Immunology | Y | 0.00188 | PTPRC;BAX;PNP;LTF;LGALS3;MFGE8;XDH;TNFRSF6B;F2;GSN;ANXA2;ANXA5;CAV1;STAT1;RAC1;ANXA1;CD36;HMGB1;STAT3;GNAQ;NCAM1;ELN | N | - | - | N | - |
| Ingestion | Miscelleneaous | Y | 0.0019 | PTPRC;PABPC4;ITGB2;ALB;CLU;HRG;TF;APOB;ITGB3;MFGE8;APOE;CP;XDH;HPRT1;VTN;CAT;PPP5C;APP;FN1;G6PD;F2;AQP1;DPP4;CALR;ANXA5;NID1;GSR;RAC1;RPLP0;RHOA;ITGAV;CDC42;LRP1;CD36;RAB7A;ITGA5;CYBB;SOD2;HMGB1;LUM;ADH1B;ALDH2;ROCK1;ITGB1;AGO1;SGCE;ARF6;GPX1;ILK;MYLK;ASPH;FBP1;MARCKS;PTK2 | N | - | - | Y | - |
| Activated T cell adhesion | Immunology | Y | 0.00203 | PTPRC;ITGB2;LGALS3;ITGB3;MSN | N | - | - | N | - |
| Renal reabsorption | Miscelleneaous | Y | 0.00224 | ALB;ACOX1;TF;HP;DAB2;XDH;CAT;AQP1;DPP4;FOLH1;PRKCSH;AHCY;CD36;GNAS;ARF6;ADD1 | N | - | - | N | - |
| Postmenopause | Miscelleneaous | Y | 0.0023 | APOB;APOE;HP;COL1A1;CAT;MMP2;TXN;F2;MAPK1;EPHX2;ELN | N | - | - | Y | - |
| Second trimester pregnancy | Miscelleneaous | Y | 0.00237 | ALB;APOA1;COL1A1;FN1;F2;GSN;VCAN;SPR;SERPINH1;ELN | N | - | - | N | - |
| Endothelial cell quantity | Vascular | Y | 0.0024 | ALB;COMP;APOA1;APOE;APP;ANXA3;F2;DPP4;SERPINF1;ACTB;GPX1;ILK;TGM2 | N | - | - | Y | - |
| Fibrin formation | Vascular | Y | 0.00248 | ALB;HRG;FGG;MFGE8;MMP2;FN1;F2;ENO1;ANXA5;P4HB;STAT3;SPARC;DPT | N | - | - | N | - |
| Osteoclast adhesion | Vascular | Y | 0.00255 | LCP1;ITGB3;VTN;MRVI1;PTK2 | N | - | - | N | - |
| Phagocyte behavior | Immunology | Y | 0.00304 | ITGB2;CAPG;PLG;LTF;LGALS3;MFGE8;HP;APP;FN1;LRP1;CYBB;HMGB1;ARF6;DYSF | N | - | - | N | - |
| Umbilical vein endothelial cell adhesion | Immunology, Vascular | Y | 0.00338 | CLU;ITGB3;VTN;FN1;F2;TGFBI;CD36;ITGB1;SPARC;DPT;FBLN5;ELN | N | - | - | N | - |
| Macrophage recognition | Immunology | Y | 0.00374 | CLU;APOB;VTN;CAT;FN1;NCL;CALR;ANXA5;CD36;TGM2 | N | - | - | N | - |
| Mast cell adhesion | Immunology | Y | 0.00375 | ALB;NAGA;LGALS3;ITGB3;VTN;F2;ITGAV;ITGB1;FES | N | - | - | N | - |
| Fibrinolysis | Vascular | Y | 0.0038 | ALB;CLEC3B;PLG;HRG;A2M;APOA1;APOB;FGG;SERPINE2;VTN;MMP2;APP;FN1;F2;ENO1;S100A10;ANXA2;SERPINF1;ANXA5;LNPEP;CAV1;HBA1;LRP1;IGF2R;GAPDH;FAP;RNPEP;PPIA;HMGB1;SERPINA3;VIM;C3;RAP1A;SPARC;DCN;SERBP1;FBLN5;ELN | N | - | - | Y | Y |
| Neutrophil retention | Immunology | Y | 0.00403 | ITGB2;RAC2;XDH;CAT;FN1;MSN;RAC1 | N | - | - | N | - |

| | | | | | | | | | |
|---|---|---|---|---|---|---|---|---|---|
| Monocyte migration | Immunology | Y | 0.0041 | ENO2;ITGB2;ACTN1;ALB;CLU;RAC2;PLG;LTF;LGALS3;GMFG;HP;CAT;HSPE1;MMP2;APP;TXN;FN1;F2;GLG1;SOD3;ENO1;GPI;DPP4;PRDX1;ANXA2;SERPINF1;CALR;MAPK3;CAV1;STAT1;TGFBI;RAC1;ANXA1;RHOA;CD9;CDC42;LRP1;CD36;YWHAZ;ITGA5;RPS19;MAPK1;PPIA;HMGB1;LAMC1;ITGAL;ROCK1;AOC3;VIM;GSK3B;ITGB1;GNAQ;HSPB1;TES;TGM2;MYLK;PTK2;DYSF;ELN | N | - | - | Y | Y |
| Vascular endothelium function | Vascular | Y | 0.0046 | ALB;RAC2;LTF;HP;AQP1;DPP4;TKT;PDE5A;CTTN;HSPA6 | N | - | - | Y | Y |
| Neutrophil adhesion | Immunology | Y | 0.00495 | PTPRC;ITGB2;ALB;RAC2;LCP1;LTF;LGALS3;APOA1;CORO1A;HPX;HP;XDH;VTN;CAT;ITIH2;MMP2;TXNRD1;APP;TXN;FN1;F2;ADK;NID1;MAPK3;CAV1;TGFBI;ANXA1;RHOA;P4HB;CD9;STX4;PRKCD;SOD2;MAPK1;PPIA;HMGB1;NT5E;ITGAL;STAT3;AOC3;ROCK1;TLN1;ITGB1;RAP1A;MYLK;TGM2;MARCKS;PTK2 | N | - | - | N | - |
| Neutrophil aggregation | Immunology | Y | 0.00505 | ITGB2;ALB;LTF;XDH;MMP2;F2;MAPK3;RAC1;MAPK1;PTK2 | N | - | - | N | - |
| NK cell mediated cytotoxicity | Immunology | Y | 0.00574 | PTPRC;ITGB2;ALB;BAX;GC;LCP1;A2M;LTF;C5;TF;LGALS3;CORO1A;LGALS3BP;ITGB3;APOE;HP;TNFRSF6B;CAT;MMP2;APP;FN1;HLA-B;F2;ARL8B;DPP4;PRDX1;CALR;MYH9;MAPK3;SET;STAT1;RAC1;EIF5A;ANXA1;P4HB;CDC42;PGM3;GRB2;PRDX2;PRKCD;CTSC;TALDO1;HSPD1;HSP90B1;MAPK1;HMGB1;CRK;HYOU1;ITGAL;DNM2;DLAT;C3;STAT3;VIM;GSK3B;PARVA;TUBB;PTPN11;KIF5B;NCAM1;HSPB1;ASPH;SVIL;DMD;SERPINH1;CRKL;NRP1;ELN | N | - | - | N | - |
| Dendritic cell behavior | Immunology | Y | 0.00575 | PLIN2;ALB;PLG;ALDH1A2;FTH1;LTF;LGALS3;APOA1;APOE;HP;TNFRSF6B;MMP2;APP;FN1;F2;FKBP1A;DPP4;MAPK3;STAT1;RAC1;ANXA1;RHOA;NDRG2;ITGAV;CDC42;FSCN1;CD36;HSPD1;PHB;MAPK1;HMGB1;STAT3;GSK3B;PTPN11;TGM2;NRP1 | N | - | - | N | - |
| Drug transport | Miscelleneaous | Y | 0.00605 | ALB;TF;APOE;CAT;MVP;APP;RPS13;CAV1;MSN;RPL23;RHOA;RDX;ELN | N | - | - | N | - |
| Endothelial cell differentiation | Vascular | Y | 0.00627 | FBLN7;ALB;LGALS3;ITGB3;HP;DAB2;VTN;TNFRSF6B;FN1;F2;PRDX1;DPP4;MAPK3;CAV1;RAC1;CDH13;RHOA;CDC42;CAPN2;MAPK1;STAT3;VIM;GSK3B;AKAP12;RAP1A;DCN;TAGLN;DDAH2;TGM2;EDF1;PTK2;NRP1 | N | - | - | N | - |
| Immune complex clearance | Immunology | Y | 0.0065 | ITGB2;HRG;C4A;LGALS3;FN1;CALR;C3 | N | - | - | N | - |
| M2 macrophage phenotype | Immunology | Y | 0.00671 | RAC2;LGALS3;APOA1;APOE;TXN;STAT1;CD36 | N | - | - | N | - |

| | | | | | | | | | |
|---|---|---|---|---|---|---|---|---|---|
| Humoral immune response | Immunology | Y | 0.0068 | PTPRC;ALB;LCP1;A2M;ALDH1A2;C4A;LTF;TF;LGALS3;APOA1;LGALS3BP;APOE;FBLN1;VTN;TNFRSF6B;CAT;PADI2;APP;SPAG9;TXN;MPZ;FN1;F2;SOD3;GPI;FABP4;NQO1;DPP4;PRDX1;NPM1;TPI1;FOLH1;CALR;RPL5;IDH1;SET;ANXA1;RPL23;STIP1;RPLP0;EIF4A3;CDC42;HSPA5;YWHAQ;EIF4A1;HNRNPA2B1;HSPD1;EEF2;HSP90B1;CYBB;GLS;MAPK1;HMGB1;HNRNPD;EEF1A1;RAP1B;C3;STAT3;IDH2;TGM2;RPL12 | N | - | - | Y | Y |
| Immunostimulation | Immunology | Y | 0.00713 | PTPRC;ITGB2;ALB;LTF;LGALS3;CTSD;MFGE8;HSPE1;DPP4;RAC1;RCN1;LRP1;CDC42;HSPD1;STAT3 | N | - | - | N | - |
| Foam cell formation | Lipid-related | Y | 0.0071 | ITGB2;PLIN2;PON3;PLG;APOA1;CORO1A;SCARB2;APOB;CTSD;APOE;XDH;CES1;UFM1;CAT;MMP2;APP;AEBP1;FN1;ACAT1;F2;FABP4;DPP4;PRDX1;CALR;MAPK3;CAV1;STAT1;RHOA;LRP1;CD36;HSPA5;GRB2;PRKCD;CAPN1;APPL1;MAPK1;PPIA;APOA1BP;DNM2;RELA;ROCK1;VIM;GSK3B;ROCK2;GPX1;HSPB1;LSS;PLIN1;PXDN | N | - | - | Y | Y |
| Luteal phase | Miscelleneaous | Y | 0.00727 | PLIN2;BAX;LTF;LGALS3;MFGE8;CP;CAT;MMP2;ANXA4;F2;ITGA1;SOD2;GNB2 | N | - | - | N | - |
| Complement activation, alternative pathway | Immunology | Y | 0.0076 | ITGB2;COMP;PLG;C4A;APP;TXN;MPZ;F2;GPI;STAT3;C3;FMOD | N | - | - | N | - |
| Mammary gland development | Miscelleneaous | Y | 0.00766 | BAX;IGFBP7;S100A4;MFGE8;XDH;MMP2;AEBP1;FN1;RPL6;NIT1;GSN;CAV1;STAT1;PRKCD;PHB2;CRK;STAT3;ITGB1;ATP2B4;RRAS2;ENAH;PTK2;KRT14;KHDRBS1 | N | - | - | N | - |
| Blood clot lysis | Vascular | Y | 0.0078 | ALB;RAC2;PLG;A2M;FGG;SERPINE2;APOE;VTN;MMP2;F2;ANXA2;LRP1;PRKCD;HMGB1;C3;PDE5A | N | - | - | Y | Y |
| Neutrophil degranulation | Immunology | Y | 0.00818 | ITGB2;ALB;RAC2;LTF;LGALS3;APOA1;FN1;MAPK3;SNAP23;ANXA1;PRKCD;MAPK1 | N | - | - | N | - |
| Neutrophil chemotaxis | Immunology | Y | 0.00828 | PTPRC;ITGB2;RAC2;GUSB;GC;C4A;LTF;TF;LGALS3;HPX;GMFG;HP;VTN;APP;SSR1;TXN;FN1;F2;SOD3;ENO1;PSMA3;NID1;MAPK3;CAV1;MSN;STAT1;RAC1;YARS;MAP2K1;RHOA;SBDS;CDC42;CAPN2;GAPDH;RPS19;MAPK1;PPIA;HMGB1;SERPINA3;RELA;ROCK1;GNA13;STAT3;GNB2;GSK3B;GNAQ;RAP1A;HSPB1;SPARC;TGM2;MARCKS;CRKL;ELN | N | - | - | N | - |

| | | | | | | | | | |
|---|---|---|---|---|---|---|---|---|---|
| Membrane damage | Miscelleneaous | Y | 0.00861 | ITGB2;ALB;CLU;BAX;NAGA;LTF;TF;LGALS3;APOB;CTSD;MFGE8;APOE;XDH;PSAP;CAT;MMP2;APP;S100A11;FN1;G6PD;NQO1;NPM1;HBB;ANXA5;HK1;MAPK3;HBA1;TPT1;ANXA1;PPIB;HSPA8;GPX5;SLC25A5;GAPDH;AIFM1;PRKCD;CAPN1;CYBB;PRDX6;MAPK1;HMGB1;ATP5O;PTRF;GSK3B;TARDBP;ACTA2;DAG1;ATP2B4;GPX1;HSPB1;DMD;DYSF | N | - | - | N | - |
| Endometrium proliferation | Miscelleneaous | Y | 0.00874 | CLU;LTF;LGALS3;NPM1;SERPINF1;CAV1;DCN | N | - | - | N | - |
| Cytolysis | Immunology | Y | 0.00944 | PTPRC;ITGB2;ALB;CLU;BAX;LCP1;A2M;C4A;FTH1;LTF;TF;LGALS3;C6;CTSD;CP;VTN;CAT;PEPD;PSME1;APP;G6PD;HLA-B;F2;ARL8B;NQO1;DPP4;CALR;ANXA5;MAPK3;LMNB1;GSR;STAT1;MAP2K1;GARS;RHOA;HSPA5;GRB2;ACTR2;GAPDH;TCEB1;HSPD1;EEF2;MAPK1;HMGB1;SERPINA3;SH3BGRL3;ACTR3;ITGAL;C3;KRT5;STAT3;GNB2;ATP2B4;PRELP;ATP5A1;NCAM1;HSPB1;ADD1;KRT10;KRT14 | N | - | - | N | - |
| Ovulation | Miscelleneaous | Y | 0.00954 | FABP5;ALB;HTRA1;PLG;A2M;IGFBP7;FLII;APOA1;CTSD;SERPINE2;APOE;XDH;HPRT1;MMP2;FN1;F2;FKBP4;ENO1;PRDX1;DPP4;ANXA2;NID1;MAPK3;CAV1;RHOA;VCAN;CDC42;PGRMC1;PRDX2;RAB7A;GDI1;HSPD1;MAPK1;DCXR;AKR1C1;HSPG2;TLN1;ITGB1;GNAQ;CBR1;HSPB1;DCN;ILK;PDE3A;FERMT2;HAPLN1 | N | - | - | N | - |
| Immunocompetent cell adhesion | Immunology | Y | 0.00956 | PTPRC;ITGB2;APP;FN1;GSK3B;PTK2 | N | - | - | N | - |
| Cellular immune response | Immunology | Y | 0.0102 | PTPRC;ITGB2;ALB;CLU;BAX;RAC2;PNP;LCP1;A2M;C4A;LTF;LGALS3;LGALS3BP;APOE;TNFRSF6B;CAT;HSPE1;MVP;APP;TXN;HSP90AA1;G6PD;HLA-DQB1;HLA-B;F2;FKBP1A;ENO1;GPI;PSME2;NQO1;DPP4;GSN;CALR;MAPK3;GSR;STAT1;RAC1;RPL23;RHOA;RPL7;P4HB;HSPA8;CDC42;LRP1;CD36;GAPDH;EIF4A1;CTSC;CRYAB;HSPD1;PARK7;HSP90B1;SOD2;MAPK1;PPIA;HSPB6;HMGB1;NDUFA13;EEF1A1;RPN1;HSPA2;RELA;STAT3;RPSA;PTPN11;ACTB;CSPG4;JUP;GPX1;DCN;HSPB1;TES;TGM2;RPL12;ATP2A3;DMD;NRP1;ELN | N | - | - | Y | Y |
| Phagocyte recognition | Immunology | Y | 0.01032 | ITGB2;LGALS3;MFGE8;VTN;CD36 | N | - | - | N | - |
| Cholesterol esterification | Lipid-related | Y | 0.01035 | ALB;A2M;APOA1;APOB;APOE;HP;CES1;APP;OSBP;ACAT1;CAV1;LRP1;HSPA5;RAB7A;MAPK1;VIM;LSS | N | - | - | N | - |
| Placenta transfer | Miscelleneaous | Y | 0.01058 | PLIN2;ALB;PLG;TF;CP;MMP2;CD36;ANXA6 | N | - | - | N | - |

| | | | | | | | | | | |
|---|---|---|---|---|---|---|---|---|---|---|
| Lymphocyte proliferation | Immunology | Y | 0.0114 | PTPRC;ITGB2;ALB;A2M;FTH1;LTF;TF;CORO1A;APOE;HP;CP;TNFRSF6B;CAT;PEPD;APP;FN1;F2;STT3A;FABP4;DPP4;ANXA2;HBB;CALR;STAT1;ANXA1;CD9;HSPD1;TALDO1;IMPDH2;MAPK1;HSP90B1;HMGB1;ITGAL;STAT3;RELA;GSK3B;ITGB1;GNAS;NCAM1 | N | - | - | N | - |
| Mucus secretion | Miscelleneaous | Y | 0.01152 | ALB;C4A;LTF;LGALS3;XDH;CAT;MAP2K1;SERPINB1;MAPK1;MARCKS | N | - | - | N | - |
| Brain microcirculation | Miscelleneaous | Y | 0.01322 | ALB;APOE;APP;F2;GPX1 | N | - | - | N | - |
| Lung permeability | Miscelleneaous | Y | 0.01351 | ITGB2;ALB;HPX;HP;XDH;F2;AQP1;CAV1;TOLLIP;RHOA;CTNNA1;HMGB1;SPARC;MYLK | N | - | - | N | - |
| Choroid blood flow | Vascular | Y | 0.01399 | HTRA1;APOE;MMP2;SERPINF1;PDE5A;RAP1A;ELN | N | - | - | N | - |
| Blood platelet phagocytosis | Immunology | Y | 0.014 | ITGB2;ITGB3;MFGE8;APP;CD36 | N | - | - | N | - |
| Thyroid function | Miscelleneaous | Y | 0.0143 | LGALS3;S100A4;APOE;TXNRD1;CALR;STAT1;GPX3;RHOA;GNAS;GNAQ;ELN | N | - | - | Y | - |
| Eosinophil function | Immunology | Y | 0.01435 | PTPRC;ITGB2;RAC2;TXN;FN1;MAPK1 | N | - | - | N | - |
| Rosetting | Immunology | Y | 0.01486 | ALB;A2M;CP;FN1;CD36 | N | - | - | N | - |
| T cell quantity | Immunology | Y | 0.0149 | PTPRC;ITGB2;ALB;LTF;LGALS3;APOE;APP;HLA-DQB1;F2;CALR;P4HB;HSPD1;BGN;ITGAL;STAT3;RELA;GPRIN1;TRIM28;KRT14 | N | - | - | Y | - |
| lymphocyte quantity | Immunology | Y | 0.01532 | ITGB2;ALB;TF;APOA1;CP;TNFRSF6B;HSPE1;SOD3;SERPINB1;ITGAL;AOC3;TES | N | - | - | N | - |
| Lipid hydrolysis | Lipid-related | Y | 0.0155 | PLIN2;ALB;APOA1;APOE;CES1;FN1;F2;MGLL;ARL8B;FABP4;ANXA5;CD36;MAPK1;PRDX6;ACSL1;GNAQ;ARF6;PLCD1;MARCKS;PLIN1 | N | - | - | N | - |
| Cell killing | Vascular, Immunology | Y | 0.01553 | ITGB2;ALB;CLU;BAX;GUSB;PNP;TF;LGALS3;LGALS3BP;MFGE8;XDH;CSTB;VTN;CES1;TNFRSF6B;CAT;MMP2;APP;TXN;FN1;F2;NQO1;NPM1;SERPINF1;CALR;APEX1;YBX1;MAPK3;SET;GSR;PPID;VDAC2;STAT1;TPT1;MAP2K1;PPIB;GSTP1;CDC42;IGF2R;HSPA9;HSPA5;AIFM1;PLS3;RECK;EEF2;SLC25A4;SOD2;MAPK1;PPIA;SERPINA3;AK3;ITGAL;C3;STAT3;PDE5A;HDLBP;PRSS1;FMOD;ARF6;CBR1;DCN;NCAM1;HSPB1;SPARC;BIN1;TGM2;PTK2;NRP1 | N | - | - | N | - |
| Th1 Th2 balance | Immunology | Y | 0.01588 | A2M;LTF;LGALS3;S100A4;HP;APP;GSN;CALR;PPIA;STAT3 | N | - | - | N | - |
| Granulocyte function | Immunology | Y | 0.0159 | ITGB2;LCP1;LTF;LGALS3;HP;FN1;DBNL;ITGAL;HSPB1;SERPINH1 | N | - | - | N | - |
| Bacterium adherence | Miscelleneaous | Y | 0.01645 | ALB;COMP;PLG;LTF;TF;FLII;SCARB2;XDH;VTN;CAT;FN1;KRT6A;NCL;F2;ENO1;PGD;GAPDH;HSPD1;SOD2;ITGA1;CRK;VIM;ITGB1;ITGB5;DCN;PTK2 | N | - | - | N | - |

| | | | | | | | | | |
|---|---|---|---|---|---|---|---|---|---|
| Vasculature development | Vascular | Y | 0.01688 | FBLN1;FN1;SERPINF1;NID1;CD36;LRP1;C14orf166;PTK2;PALLD;NRP1 | N | - | - | N | - |
| T cell death | Immunology | Y | 0.01694 | PTPRC;BAX;LTF;TF;LGALS3;CTSD;CAT;FN1;SNAP23;AIFM1;MAPK1;STAT3;MAT2B | N | - | - | N | - |
| Fetus growth | Miscelleneaous | Y | 0.01697 | PTPRC;ALB;C5;LGALS3;APOA1;APOE;CP;CAT;TXN;F2;ACLY;DPP4;ALDH9A1;GSR;MAPK3;MTHFD1;LAMA5;IGF2R;MAPK1;NDRG1;GOT2;PDE5A;ACP1;GNAS;ASPH | N | - | - | N | - |
| First trimester pregnancy | Miscelleneaous | Y | 0.017 | ALB;BAX;PLG;LTF;TF;APOA1;HEXA;APOB;ITGB3;SERPINE2;CAT;MMP2;FN1;F2;AQP1;CALR;MAPK3;STAT1;CACYBP;GAPDH;YWHAQ;BGN;SOD2;MAPK1;PPIA;VASP;STAT3;C3;RELA;ROCK1;HSPB1;ILK;KRT10;TGM2;LTA4H;SERPINH1;FBLN5 | N | - | - | N | - |
| Intestine function | Miscelleneaous | Y | 0.0176 | ALB;LTF;XDH;APP;HLA-DQB1;NQO1;DPP4;MAPK3;CDX1;PRDX4;HSP90B1 | N | - | - | Y | - |
| Blast transformation | Immunology | Y | 0.01777 | ALB;A2M;FN1;F2;DPP4;SPR | N | - | - | N | - |
| Sodium reabsorption | Miscelleneaous | Y | 0.01812 | ALB;LGALS3;HEXA;SERPINE2;PPP5C;AQP1;DPP4;ATP1A1;MAPK3;RAC1;P4HB;ARHGDIA;AHCY;MAPK1;CTTN;ADD1 | N | - | - | N | - |
| Virus attachment | Miscelleneaous | Y | 0.01829 | PTPRC;ALB;SDCBP;LTF;APOA1;CTBP1;SCARB2;MFGE8;APOE;VTN;APP;FN1;NCL;F2;ILF3;FKBP4;DPP4;ANXA2;ATP1A1;CAV1;RAC1;ANXA1;P4HB;ITGAV;CD36;HSPA5;GRB2;RAB7A;PPIA;HSP90B1;HSPG2;VIM;DAG1;ARCN1;RPSA;ITGB1;SERPINH1 | N | - | - | N | - |
| Capillary permeability | Vascular | Y | 0.0183 | ITGB2;ALB;COMP;HNMT;PLG;TF;APOB;ITGB3;HPX;XDH;VTN;CAT;MMP2;APP;FN1;F2;PLXDC2;SERPINF1;MAPK3;CAV1;COL15A1;ARHGDIA;RHOA;PRKCD;RDX;SOD2;PTGIS;MAPK1;HMGB1;HSPG2;STAT3;ROCK1;ROCK2;PDE5A;ITGB1;RAP1A;HSPB1;MYLK;DTNA;PTK2;ELN | N | - | - | Y | Y |
| Cell adhesion | Immunology, Vascular | N | - | - | Y | 3.8E-21 | HTRA1;GMFG;CCDC80;COMP;LTF;FLOT2;HRG;RAC2;MFGE8;PLG;SDCBP;HP;ALB;CTSD;C4A;XDH;FLII;CP;FGG;FBLN7;IGFBP7;TF;APOE;FABP5;CLU;FABP7;CORO1A;DAB2;PTPRC;CTHRC1;LGALS3;LGALS3BP;ITGB2;BAX;LCP1;APOA1;SERPINE2;ACTN1;GUSB;SCARB2;ITIH1;FBLN1;HPX;A2M;S100A4;ITGB3 | Y | - |

| | | | | | | | | | |
|---|---|---|---|---|---|---|---|---|---|
| Cell differentiation | Immunology; Vascular; Lipid-related | N | - | - | Y | 8.2E-20 | GMFG;CCDC80;LTF;COMP;DDX6;MFGE8;PLG;ALB;CP;TF;ALDH1A2;IGFBP7;APOE;FABP5;CLU;DAB2;ETHE1;CTHRC1;LGALS3;APMAP;BAX;TANC1;ACTN1;CTSH;FBLN1;A2M;HPX;S100A4;CA2;PABPC4;HTRA1;APOB;FLOT2;HNMT;RAC2;CLEC3B;SDCBP;HP;CTSD;COL1A1;C4A;XDH;FLII;CSTB;FBLN7;CTBP1;FABP7;TBC1D10C;CAPG;ACOX1;PLIN2;CORO1A;PTPRC;AOX1;ENO2;LGALS3BP;ITGB2;NMT1;APOA1;SERPINE2;PNP;HPRT1;ITGB3;FTH1;GC | Y | - |
| Cell behavior | Miscelleneaous | N | - | - | Y | 1.3E-19 | GMFG;LTF;COMP;MFGE8;PLG;ALB;CP;IGFBP7;TF;ALDH1A2;APOE;FABP5;CLU;DAB2;LGALS3;BAX;LCP1;ACTN1;GUSB;CTSH;FBLN1;HPX;S100A4;A2M;CA2;HTRA1;APOB;FLOT2;RAC2;HP;TWF1;C4A;CTSD;XDH;CTBP1;FABP7;PLIN2;CORO1A;PTPRC;ENO2;LGALS3BP;ITGB2;APOA1;SERPINE2;PNP;ITGB3;FTH1;GC | Y | - |
| Non-selective vesicle endocytosis | Lipid-related | N | - | - | Y | 3.7E-19 | APOB;LTF;NAPA;FLOT2;RAC2;MFGE8;SDCBP;PLG;HP;TWF1;ALB;TWF2;CTSD;EHD4;XDH;CTBP1;TF;APOE;TBC1D10C;CLU;DAB2;PTPRC;LGALS3;ITGB2;HEXA;BAX;APOA1;SERPINE2;ACTN1;GUSB;SCARB2;A2M;HPX;ITGB3;GC | Y | - |
| Immunity | Immunology | N | - | - | Y | 4.3E-18 | HTRA1;APOB;LTF;FLOT2;HRG;MFGE8;RAC2;SDCBP;PLG;HP;ALB;C4A;CTSD;CP;CSTB;TF;IGFBP7;APOE;FABP5;CLU;PTPRC;LGALS3;LGALS3BP;ITGB2;BAX;LCP1;APOA1;SERPINE2;CTSH;A2M;HPX;S100A4;FTH1;GC | Y | - |
| Angiogenesis | Vascular | N | - | - | Y | 7.3E-18 | CA2;HTRA1;GMFG;APOB;LTF;COMP;FLOT2;HRG;RAC2;MFGE8;SDCBP;PLG;HP;TWF1;ALB;COL1A1;CTSD;XDH;CP;CTBP1;FGG;FBLN7;TF;ALDH1A2;IGFBP7;APOE;CLU;FGB;DAB2;PTPRC;CTHRC1;LGALS3;LGALS3BP;ITGB2;BAX;APOA1;SERPINE2;GUSB;CTSH;FBLN1;ITIH1;HPX;A2M;S100A4;ITGB3;GC | Y | Y |
| Regeneration | Miscelleneaous | N | - | - | Y | 1.4E-17 | C6;LTF;NAPA;COMP;FLOT2;MFGE8;PLG;SDCBP;HP;ALB;COL1A1;FLII;TF;ALDH1A2;APOE;FABP5;CLU;ATP6V1E1;ACOX1;PTPRC;LGALS3;ENO2;ITGB2;BAX;TANC1;LCP1;APOA1;SERPINE2;GUSB;SCARB2;HPRT1;A2M;S100A4;HPX;ITGB3 | Y | - |

| | | | | | | | | | |
|---|---|---|---|---|---|---|---|---|---|
| Cell invasion | Immunology, Vascular | N | - | - | Y | 2.5E-17 | CA2;GPC6;HTRA1;GMFG;CCDC80;LTF;NAPA;COMP;FLOT2;MFGE8;RAC2;PLG;SDCBP;ALB;COL1A1;CTSD;FLII;XDH;CTBP1;TF;IGFBP7;APOE;FABP5;CLU;FABP7;CAPG;PTPRC;DAB2;CTHRC1;LGALS3;LGALS3BP;ITGB2;BAX;LCP1;SERPINE2;ACTN1;PNP;GUSB;CTSH;FBLN1;HPX;A2M;S100A4;ITGB3 | Y | Y |
| Innate immune response | Immunology | N | - | - | Y | 3.4E-17 | APOB;SSC5D;LTF;HRG;MFGE8;RAC2;PLG;HP;ALB;C4A;CTSD;XDH;CP;CSTB;TF;APOE;CLU;FGB;PTPRC;LGALS3;ITGB2;BAX;LCP1;APOA1;PNP;C7;CTSH;SCARB2;A2M;ITGB3;GC | Y | - |
| Inflammatory response | Immunology | N | - | - | Y | 1.3E-16 | C6;APOB;LTF;C5;HRG;MFGE8;RAC2;PLG;HP;ALB;C4A;CTSD;FLII;XDH;CP;CSTB;FGG;IGFBP7;TF;APOE;FABP5;CLU;CAPG;CORO1A;PTPRC;DAB2;LPCAT2;LGALS3;ITGB2;BAX;LCP1;APOA1;SERPINE2;GUSB;C7;CTSH;SCARB2;A2M;S100A4;HPX;ITGB3;GC | Y | Y |
| Immune response | Immunology | N | - | - | Y | 3.6E-16 | C6;PON3;APOB;C5;LTF;HRG;MFGE8;RAC2;PLG;SDCBP;HP;ALB;C4A;CTSD;XDH;CP;CSTB;TF;ALDH1A2;APOE;FABP5;CLU;CES2;CORO1A;FGB;PTPRC;LGALS3;ITGB2;LGALS3BP;BAX;LCP1;APOA1;PNP;GUSB;SCARB2;HPX;A2M;S100A4;ITGB3;FTH1;GC | Y | - |
| Cell survival | Immunology | N | - | - | Y | 3.8E-16 | CA2;HTRA1;NAPA;LTF;COMP;FLOT2;RAC2;MFGE8;PLG;SDCBP;HP;ALB;CTSD;COL1A1;XDH;FLII;CSTB;CTBP1;IGFBP7;TF;APOE;FABP5;FABP7;CLU;PLIN2;CORO1A;PTPRC;DAB2;CTHRC1;ENO2;LGALS3;ITGB2;BAX;LCP1;APOA1;SERPINE2;ACTN1;PNP;HPRT1;A2M;HPX;S100A4;ITGB3;RPL35A;FTH1;GC | Y | - |
| Cell formation | Immunology; Lipid-related | N | - | - | Y | 4.0E-16 | GMFG;LTF;C5;MFGE8;RAC2;SDCBP;PLG;ALB;COL1A1;CTSD;XDH;FLII;ALDH1A2;IGFBP7;TF;APOE;FABP5;CLU;PLIN2;CORO1A;PTPRC;DAB2;CTHRC1;LGALS3;ENO2;RPL36A;ITGB2;BAX;LCP1;APOA1;SERPINE2;ACTN1;SCARB2;FBLN1;HPRT1;A2M;S100A4;ITGB3;FTH1 | Y | - |
| Cell motility | Immunology | N | - | - | Y | 4.8E-16 | GPC6;CA2;HTRA1;GMFG;LTF;MFGE8;RAC2;SDCBP;PLG;TWF1;ALB;CTSD;FLII;CSTB;TF;APOE;FABP5;FABP7;CLU;CAPG;CORO1A;DAB2;PTPRC;CTHRC1;LGALS3;ITGB2;LGALS3BP;LCP1;APOA1;ACTN1;C7;FBLN1;S100A4;ITGB3 | Y | - |

| | | | | | | | | | |
|---|---|---|---|---|---|---|---|---|---|
| Chemotaxis | Immunology | N | - | - | Y | 1.8E-15 | GMFG;APOB;COMP;LTF;FLOT2;RAC2;MFGE8;PLG;SDCBP;HP;ALB;C4A;CTSD;TF;APOE;FABP5;CLU;CORO1A;PTPRC;LGALS3;ITGB2;LCP1;APOA1;ACTN1;GUSB;HPX;S100A4;A2M;ITGB3;FTH1;GC | Y | - |
| Phagocytosis | Immunology | N | - | - | Y | 2.5E-15 | PABPC4;GMFG;C5;LTF;HRG;RAC2;MFGE8;PLG;HP;ALB;C4A;COL1A1;CP;TF;APOE;FABP7;CLU;CAPG;CORO1A;PTPRC;LGALS3;ITGB2;LCP1;APOA1;GUSB;SCARB2;A2M;HPX;ITGB3;FTH1 | Y | Y |
| Actin organization | Miscelleneaous | N | - | - | Y | 4E-15 | GMFG;LTF;FLOT2;RAC2;PLG;SDCBP;HP;TWF1;ALB;TWF2;CTSD;FLII;XDH;TF;CAPG;CORO1A;PTPRC;CTHRC1;LGALS3;ENO2;ITGB2;LCP1;APOA1;ACTN1;HPX;S100A4;ITGB3;GC | N | - |
| Lipid metabolism | Lipid-related | N | - | - | Y | 6.3E-15 | CA2;HTRA1;PON3;APOB;LTF;DDX6;HRG;PLG;HP;ALB;COL1A1;C4A;CTBP1;TF;APOE;FABP5;CLU;FABP7;CES2;ACOX1;PLIN2;AOX1;ENO2;APMAP;DECR1;APOA1;A2M;ITGB3;GC | Y | Y |
| Internalization | Lipid-related | N | - | - | Y | 3.6E-14 | PABPC4;APOB;LTF;FLOT2;HRG;RAC2;MFGE8;SDCBP;PLG;HP;TWF1;ALB;EHD4;CP;TF;APOE;CLU;DAB2;PTPRC;LGALS3;ITGB2;NAGA;BAX;APOA1;SERPINE2;ACTN1;GUSB;SCARB2;A2M;ITGB3;FTH1 | Y | - |
| Apoptosis | Vascular, Immunology | N | - | - | Y | 9.0E-14 | CCDC80;COMP;LTF;C5;HRG;DDX6;MFGE8;PLG;ALB;CP;TF;IGFBP7;ALDH1A2;APOE;FABP5;CLU;ETHE1;DAB2;CTHRC1;LGALS3;BAX;ACTN1;GUSB;CTSH;FBLN1;HPX;A2M;S100A4;CA2;HTRA1;PON3;APOB;NAPA;FLOT2;RAC2;SDCBP;CLEC3B;GLB1;HP;C4A;COL1A1;CTSD;XDH;FLII;CTBP1;CSTB;FGG;CAPG;CES2;ACOX1;PLIN2;CORO1A;PTPRC;ENO2;LGALS3BP;ITGB2;RPL36A;NMT1;APOA1;SERPINE2;PNP;SCARB2;ITGB3;RPL35A;FTH1;GC | Y | Y |
| Cell migration | Immunology, Vascular | N | - | - | Y | 1.0E-13 | GPC6;HTRA1;GMFG;CCDC80;COMP;LTF;FLOT2;HRG;RAC2;MFGE8;PLG;SDCBP;HP;TWF1;ALB;CTSD;COL1A1;FLII;XDH;CTBP1;CSTB;TF;IGFBP7;APOE;FABP5;FABP7;CLU;CAPG;PTPRC;DAB2;CTHRC1;LGALS3;ENO2;ITGB2;LGALS3BP;LCP1;APOA1;SERPINE2;ACTN1;CTSH;FBLN1;HPRT1;HPX;A2M;S100A4;ITGB3;FTH1 | Y | - |

| | | | | | | | | | |
|---|---|---|---|---|---|---|---|---|---|
| Cell development | Immunology, Lipid-related | N | - | - | Y | 2.2E-13 | CA2;HTRA1;LTF;C5;COMP;DDX6;MFGE8;RAC2;SDCBP;HP;ALB;XDH;CTBP1;ALDH1A2;TF;APOE;FABP7;CLU;PLIN2;CORO1A;PTPRC;DAB2;LGALS3;ENO2;ITGB2;BAX;APOA1;ACTN1;PNP;FBLN1;S100A4;ITGB3;FTH1 | Y | - |
| Cell proliferation | Vascular | N | - | - | Y | 6.7E-13 | CCDC80;COMP;LTF;HRG;DDX6;MFGE8;PLG;ALB;CP;IGFBP7;TF;ALDH1A2;APOE;FABP5;CLU;ATP6V1E1;DAB2;CTHRC1;LGALS3;BAX;LCP1;ACTN1;GUSB;CTSH;FBLN1;S100A4;HPX;A2M;CA2;HTRA1;PON3;APOB;NAPA;FLOT2;HNMT;RAC2;SDCBP;HP;COL1A1;CTSD;XDH;FLII;CTBP1;CSTB;FABP7;CAPG;CES2;ACOX1;PLIN2;CORO1A;PTPRC;ENO2;RPL36A;ITGB2;LGALS3BP;HEXA;NMT1;DECR1;APOA1;SERPINE2;PNP;HPRT1;ITGB3;RPL35A;FTH1;GC | Y | Y |
| Aging | Miscelleneaous | N | - | - | Y | 6.8E-13 | HTRA1;APOB;COMP;RAC2;MFGE8;HP;ALB;CTSD;COL1A1;XDH;CP;FGG;TF;IGFBP7;APOE;FABP5;CLU;ACOX1;PLIN2;PTPRC;CTHRC1;LGALS3;BAX;APOA1;GUSB;A2M;HPX | Y | - |
| Adipocyte differentiation | Lipid-related | N | - | - | Y | 2E-12 | CTHRC1;APMAP;LGALS3;LGALS3BP;CCDC80;LTF;APOA1;CLEC3B;ALB;CTSD;XDH;FLII;CP;IGFBP7;TF;A2M;APOE;FABP5;ACOX1;PLIN2;FTH1 | N | - |
| Blood clotting | Vascular | N | - | - | Y | 3.1E-12 | DAB2;LGALS3;ITGB2;APOB;LTF;HRG;APOA1;MFGE8;PLG;HP;SERPINE2;ALB;CTSD;C4A;XDH;FGG;TF;A2M;HPX;ITGB3;GC;FGB | Y | Y |
| Synaptogenesis | Miscelleneaous | N | - | - | Y | 5E-12 | PTPRC;GPC6;ITGB2;BAX;COMP;LCP1;FLOT2;RAC2;SDCBP;GLB1;SERPINE2;ACTN1;ALB;CTSD;TF;APOE;FABP7;CLU;CORO1A | N | - |
| Cell death | Vascular | N | - | - | Y | 1.0E-11 | LTF;COMP;MFGE8;PLG;ALB;CP;IGFBP7;TF;APOE;CLU;DAB2;LGALS3;BAX;LCP1;ACTN1;GUSB;CTSH;FBLN1;S100A4;A2M;HPX;CA2;C6;HTRA1;PON3;APOB;NAPA;FLOT2;RAC2;HP;AARS;COL1A1;CTSD;XDH;CSTB;PLIN2;CORO1A;PTPRC;AOX1;ENO2;ITGB2;NMT1;APOA1;SERPINE2;PNP;C7;HPRT1;ITGB3;RPL35A;FTH1;GC | Y | - |
| Platelet activation | Vascular, Immunology | N | - | - | Y | 1.1E-11 | DAB2;PTPRC;CA2;LGALS3;ITGB2;APOB;C5;HRG;APOA1;PLG;SERPINE2;ACTN1;ALB;FGG;TF;SCARB2;APOE;ITGB3;CLU | Y | Y |

| | | | | | | | | | |
|---|---|---|---|---|---|---|---|---|---|
| Cell phenotype | Immunology, Vascular | N | - | - | Y | 1.2E-11 | HTRA1;COMP;LTF;FLOT2;HRG;DDX6;RAC2;SDCBP;PLG;HP;TWF1;ALB;CTBP1;IGFBP7;APOE;FABP7;CLU;CAPG;PLIN2;DAB2;PTPRC;CTHRC1;LGALS3;ITGB2;BAX;APOA1;SERPINE2;ACTN1;C7;HPRT1;FBLN1;A2M;S100A4;ITGB3 | Y | - |
| Proteolysis | Vascular | N | - | - | Y | 2.1E-11 | HTRA1;LTF;COMP;FLOT2;PLG;CLEC3B;HP;ALB;CTSD;XDH;CSTB;APOE;CLU;LGALS3;BAX;LCP1;APOA1;SERPINE2;ACTN1;GUSB;CTSH;SCARB2;FBLN1;HPX;S100A4;A2M;ITGB3 | Y | Y |
| Cell spreading | Vascular | N | - | - | Y | 2E-11 | PTPRC;DAB2;LGALS3;ITGB2;LGALS3BP;LTF;FLOT2;RAC2;APOA1;PLG;SDCBP;SERPINE2;ACTN1;ALB;FLII;IGFBP7;FBLN1;A2M;ITGB3 | N | - |
| Wound healing | Vascular | N | - | - | Y | 3.7E-11 | COMP;LTF;HRG;MFGE8;RAC2;PLG;CLEC3B;HP;ALB;COL1A1;CTSD;XDH;FLII;IGFBP7;FABP5;CLU;CAPG;PTPRC;CTHRC1;LGALS3;ITGB2;BAX;FBLN1;A2M;S100A4;ITGB3 | Y | Y |
| Detoxification (process) | Miscelleneaous | N | - | - | Y | 6E-11 | ETHE1;AOX1;DHRS4;LTF;HRG;APOA1;HP;ALB;XDH;GUSB;CP;TF;HPX;APOE;ITGB3;CAPG;CES2;FTH1 | N | - |
| Cell growth | Vascular | N | - | - | Y | 7.2E-11 | CCDC80;LTF;DDX6;HRG;MFGE8;PLG;ALB;CP;TF;ALDH1A2;IGFBP7;APOE;FABP5;CLU;DAB2;CTHRC1;LGALS3;BAX;GUSB;FBLN1;S100A4;A2M;HPX;CA2;HTRA1;NAPA;FLOT2;RAC2;SDCBP;HP;AARS;COL1A1;CTSD;XDH;FLII;CTBP1;FGG;FABP7;PLIN2;PTPRC;ENO2;ITGB2;LGALS3BP;NMT1;APOA1;SERPINE2;PNP;ITGB3;FTH1 | Y | Y |
| Lumen formation | Miscelleneaous | N | - | - | Y | 8E-11 | DAB2;CTHRC1;LGALS3;HTRA1;ITGB2;GMFG;COMP;LTF;HRG;APOA1;PLG;HP;SERPINE2;ALB;COL1A1;CTSD;XDH;FBLN7;ALDH1A2;IGFBP7;S100A4;ITGB3;CLU | N | - |
| T-cell activation | Immunology | N | - | - | Y | 8.6E-11 | PTPRC;LGALS3;LGALS3BP;ITGB2;BAX;APOB;LTF;LCP1;FLOT2;HRG;APOA1;MFGE8;RAC2;HP;ALB;ACTN1;TF;HPRT1;S100A4;APOE;FTH1;CORO1A | Y | Y |
| Tissue remodeling | Vascular | N | - | - | Y | 1.1E-10 | CTHRC1;LGALS3;MFGE8;PLG;CLEC3B;SERPINE2;HP;CTSD;CTSH;FBLN1;S100A4;HPX;A2M;CLU | Y | Y |

| | | | | | | | | | | |
|---|---|---|---|---|---|---|---|---|---|---|
| Transendothelial migration | Immunology | N | - | - | Y | 1.5E-10 | PTPRC;LGALS3;LGALS3BP;HTRA1;ITGB2;APOB;RAC2;APOA1;PLG;HP;ALB;XDH;A2M;S100A4;ITGB3;FGB | Y | Y |
| Receptor mediated endocytosis | Lipid-related | N | - | - | Y | 2.0E-10 | DAB2;TF;APOB;HPX;LTF;A2M;APOE;CLU;HP;TWF1;ALB | Y | - |
| Embryonal development | Miscelleneaous | N | - | - | Y | 2.1E-10 | HTRA1;GMFG;APOB;LTF;DDX6;PLG;HP;ALB;CTSD;FLII;CP;CTBP1;TF;IGFBP7;ALDH1A2;APOE;FABP7;CLU;ATP6V1E1;ETHE1;DAB2;LGALS3;NMT1;BAX;TANC1;APOA1;SERPINE2;A2M | Y | - |
| Cell quantity | Immunology, Vascular | N | - | - | Y | 2.1E-10 | CA2;NAPA;LTF;FLOT2;HRG;GLB1;HP;ALB;COL1A1;C4A;CTSD;XDH;CP;CTBP1;ALDH1A2;IGFBP7;TF;APOE;FABP5;CLU;PLIN2;DAB2;PTPRC;CTHRC1;LGALS3;ITGB2;BAX;LCP1;APOA1;SERPINE2;FBLN1;A2M;S100A4;HPX;FTH1;GC | Y | - |
| Mammary gland involution | Miscelleneaous | N | - | - | Y | 2E-10 | DAB2;CTSD;XDH;BAX;LTF;CLU;MFGE8;PLG | N | - |
| Developmental process | Miscelleneaous | N | - | - | Y | 3E-10 | PTPRC;LGALS3;HTRA1;PABPC4;ITGB2;BAX;COMP;RAC2;PLG;SERPINE2;HP;TWF1;ACTN1;CTSD;PNP;FLII;TF;ALDH1A2;A2M;APOE;FABP7;ITGB3 | N | - |
| Lipid transport | Lipid-related | N | - | - | Y | 3.8E-10 | APOB;LTF;APOA1;MFGE8;PLG;ALB;SCARB2;TF;APOE;FABP5;FABP7;CLU;PLIN2 | Y | Y |
| Cell phagocytosis | Immunology | N | - | - | Y | 4E-10 | LGALS3;ITGB2;LTF;HRG;MFGE8;RAC2;HP;ALB;C4A;TF;A2M;APOE;ITGB3;CORO1A | N | - |
| ECM degradation | Vascular | N | - | - | Y | 4.5E-10 | HTRA1;COMP;APOA1;PLG;SERPINE2;HP;CTSD;GUSB;CTSH;A2M;S100A4;HPX;CLU | Y | Y |
| Superoxide anion generation | Stress | N | - | - | Y | 4.5E-10 | PTPRC;LGALS3;ITGB2;DHRS4;BAX;PON3;LTF;FLOT2;RAC2;APOA1;HP;ALB;COL1A1;CTSD;XDH;GUSB;CP;TF;APOE;A2M;ITGB3;ACOX1;FTH1 | Y | Y |
| Epithelial to mesenchymal transition | Miscelleneaous | N | - | - | Y | 5E-10 | HTRA1;GMFG;FLOT2;HRG;MFGE8;PLG;SDCBP;TWF1;ALB;COL1A1;XDH;CTBP1;IGFBP7;CLU;DAB2;CTHRC1;LGALS3;BAX;APOA1;ACTN1;FBLN1;S100A4;ITGB3;FTH1 | N | - |
| Antigen processing and presentation | Immunology | N | - | - | Y | 8.5E-10 | LGALS3;ITGB2;BAX;LTF;LCP1;APOA1;RAC2;PLG;HP;ALB;CTSD;XDH;CTSH;A2M;APOE;PLIN2 | Y | - |

| | | | | | | | | | | |
|---|---|---|---|---|---|---|---|---|---|---|
| T-cell response | Immunology | N | - | - | Y | 1.4E-09 | PTPRC;LGALS3;ITGB2;APOB;LTF;LCP1;APOA1;HP;ALB;CTSD;PNP;TF;APOE;A2M;ITGB3;CLU;FTH1;GC;CORO1A | Y | - |
| Morphogenesis | Miscelleneaous | N | - | - | Y | 2E-09 | DAB2;CA2;CTHRC1;LGALS3;ITGB2;BAX;APOB;PLG;SDCBP;TWF1;ALB;CTSD;CTSH;TF;ALDH1A2;FBLN1;S100A4;APOE;A2M;HPX;CLU;ITGB3 | N | - |
| T cell migration | Immunology | N | - | - | Y | 3E-09 | PTPRC;LGALS3;ITGB2;GMFG;LCP1;RAC2;APOA1;SDCBP;ACTN1;ALB;ITGB3;CORO1A | N | - |
| Cell contact | Immunology, Vascular | N | - | - | Y | 3E-09 | DAB2;PTPRC;LGALS3;ITGB2;LGALS3BP;LTF;LCP1;DECR1;APOA1;PLG;CTSD;S100A4;CLU;ITGB3 | N | - |
| Cytoskeleton organization and biogenesis | Miscelleneaous | N | - | - | Y | 3E-09 | DAB2;CTHRC1;LGALS3;ITGB2;GMFG;LCP1;FLOT2;HRG;RAC2;MFGE8;SDCBP;HP;ALB;ACTN1;FLII;S100A4;APOE;ITGB3;CAPG;CORO1A | N | - |
| Colony formation | Miscelleneaous | N | - | - | Y | 4E-09 | CA2;CCDC80;COMP;LTF;RAC2;SDCBP;ALB;CTSD;ALDH1A2;TF;IGFBP7;FABP5;CLU;FABP7;DAB2;CTHRC1;ENO2;LGALS3;RPL36A;BAX;SERPINE2;PNP;FBLN1;S100A4;A2M;ITGB3;FTH1 | N | - |
| Viral entry | Miscelleneaous | N | - | - | Y | 4E-09 | PTPRC;LGALS3;APOB;LTF;APOA1;SDCBP;ALB;CP;SCARB2;APOE;CLU;ITGB3;PLIN2;GC | N | - |
| Viral reproduction | Miscelleneaous | N | - | - | Y | 4E-09 | APOB;LTF;DDX6;MFGE8;PLG;HP;ALB;CP;CTBP1;CSTB;APOE;CLU;PLIN2;PTPRC;ENO2;LGALS3;ITGB2;LGALS3BP;NMT1;BAX;APOA1;ACTN1;SCARB2;FTH1 | N | - |
| Endothelial cell adhesion | Immunology, Vascular | N | - | - | Y | 4.2E-09 | DAB2;LGALS3;ITGB2;LTF;HRG;APOA1;MFGE8;PLG;ALB;XDH;CP;APOE;CLU;ITGB3 | Y | Y |
| Non-selective vesicle exocytosis | Lipid-related, Immunology | N | - | - | Y | 4E-09 | DAB2;PTPRC;ITGB2;GMFG;BAX;LTF;NAPA;HNMT;MFGE8;RAC2;APOA1;PLG;ALB;CTSD;C4A;CTBP1;TF;S100A4;APOE;CORO1A | N | - |
| Neuronal death | Miscelleneaous | N | - | - | Y | 4.4E-09 | APOB;LTF;MFGE8;PLG;CLEC3B;ALB;CTSD;XDH;CP;CSTB;TF;APOE;CLU;LGALS3;ENO2;BAX;SERPINE2;GUSB;CTSH;HPRT1;S100A4;HPX;A2M;GC | Y | - |
| Bone resorption | Miscelleneaous | N | - | - | Y | 4.4E-09 | CA2;PTPRC;CTHRC1;LGALS3;LTF;APOA1;RAC2;MFGE8;HP;ALB;CTSD;COL1A1;XDH;GUSB;CSTB;S100A4;ITGB3;GC | Y | - |

| | | | | | | | | | |
|---|---|---|---|---|---|---|---|---|---|
| Cell localization | Miscelleneaous | N | - | - | Y | 5E-09 | PTPRC;DAB2;LGALS3;NMT1;BAX;NAPA;LCP1;FLOT2;DDX6;RAC2;SDCBP;TWF1;ACTN1;ALB;SCARB2;TF;HPX;APOE;ITGB3;CAPG;FTH1;CORO1A | N | - |
| Virulence | Miscelleneaous | N | - | - | Y | 5E-09 | AOX1;HTRA1;ITGB2;HEXA;NMT1;BAX;APOB;LTF;PLG;CLEC3B;ALB;XDH;CP;TF;HPRT1;A2M;CLU | N | - |
| Lipid storage | Lipid-related | N | - | - | Y | 6.9E-09 | LGALS3;HEXA;PON3;BAX;CCDC80;APOB;LTF;MFGE8;APOA1;PLG;ALB;XDH;CTBP1;ALDH1A2;TF;SCARB2;APOE;FABP5;CLU;FABP7;ACOX1;PLIN2 | Y | Y |
| Leukocyte cell adhesion | Immunology | N | - | - | Y | 7.1E-09 | PTPRC;LGALS3;ITGB2;COMP;LTF;LCP1;APOA1;PLG;HP;ALB;XDH;CP;TF;A2M;ITGB3 | Y | Y |
| Fibroblast migration | Vascular | N | - | - | Y | 7.3E-09 | DAB2;LGALS3;LTF;PLG;HP;CTSD;FLII;IGFBP7;FBLN1;APOE;S100A4;ITGB3 | Y | Y |
| Hemato-encephalic barrier | Miscelleneaous | N | - | - | Y | 7.6E-09 | ENO2;LGALS3;APOB;LTF;HNMT;APOA1;PLG;SERPINE2;ACTN1;ALB;XDH;PNP;CP;TF;APOE;S100A4;CLU | Y | - |
| Blood vessel development | Vascular | N | - | - | Y | 8.2E-09 | PTPRC;BAX;APOB;APOA1;PLG;SERPINE2;HP;ALB;COL1A1;XDH;IGFBP7;FBLN1;APOE;CLU | Y | - |
| DNA recombination | Miscelleneaous | N | - | - | Y | 9E-09 | PTPRC;CA2;BAX;APOB;NAPA;LTF;DDX6;HP;ALB;COL1A1;CTSD;GUSB;CTBP1;HPRT1;S100A4;FABP7;ITGB3;ACOX1;CORO1A | N | - |
| Extracellular matrix polymerization | Vascular | N | - | - | Y | 9.5E-09 | LGALS3;HTRA1;CCDC80;COMP;CLEC3B;SERPINE2;ALB;COL1A1;IGFBP7;FBLN1;APOE;S100A4 | Y | - |
| Immune system function | Immunology | N | - | - | Y | 1.2E-08 | PTPRC;LGALS3;ITGB2;LTF;RAC2;APOA1;MFGE8;HP;ALB;CTSD;C4A;PNP;CP;IGFBP7;APOE;HPX;FTH1;GC | Y | - |
| Adipogenesis | Lipid-related | N | - | - | Y | 2E-08 | AOX1;CTHRC1;APMAP;LGALS3;HTRA1;BAX;CCDC80;LTF;SDCBP;PLG;ALB;CTSD;XDH;CTSH;TF;APOE;FABP5;ACOX1;PLIN2 | N | - |
| Monocyte adhesion | Immunology | N | - | - | Y | 1.7E-08 | PTPRC;LGALS3;ITGB2;LGALS3BP;GMFG;APOB;LTF;APOA1;PLG;SERPINE2;ALB;XDH;APOE;ITGB3 | Y | Y |
| Cell damage | Stress | N | - | - | Y | 1.9E-08 | CA2;APOB;C5;LTF;MFGE8;PLG;HP;ALB;CTSD;XDH;CP;CSTB;TF;APOE;CLU;AOX1;LGALS3;ENO2;ITGB2;BAX;APOA1;ACTN1;PNP;GUSB;A2M;HPX;GC | Y | Y |

| | | | | | | | | | |
|---|---|---|---|---|---|---|---|---|---|
| Hemostasis | Immunology | N | - | - | Y | 2.0E-08 | DAB2;LGALS3;COMP;HRG;MFGE8;APOA1;PLG;ALB;XDH;FBLN1;A2M;ITGB3 | Y | - |
| Liver development | Miscelleneaous | N | - | - | Y | 2E-08 | LGALS3;ITGB2;APOB;C5;HP;ALB;IGFBP7;ALDH1A2;A2M;S100A4;ACOX1;PLIN2 | N | - |
| Cell aggregation | Miscelleneaous | N | - | - | Y | 2.3E-08 | PTPRC;LGALS3;ITGB2;LGALS3BP;SDCBP;PLG;HP;ALB;CP;IGFBP7;TF;A2M;CLU;ITGB3;CAPG | Y | - |

Table S1. Summary of enriched cell process subnetworks between BIAHR and H protein extracts. The 178 subnetworks found enriched with either SNEA or FSNE test or both are reported. Subnetworks present in the top 100 in both tests are displayed first ranked from the lower to the higher SNEA p-value. The existence or not of functional association relations between cell process and atherosclerosis in Pathway Studio is reported. Cell processes selected for Figure 3a are identified.
