# Supplementary material for: An innovative flow cytometry method to screen human scFv-phages selected by in vivo phage-display in an animal model of atherosclerosis
Source: Sci Rep. 2018 Oct 9;8:15016. doi: 10.1038/s41598-018-33382-2 (PMC6177473; doi:10.1038/s41598-018-33382-2)

An innovative flow cytometry method to screen human scFv-phages selected by *in vivo* phage-display in an animal model of atherosclerosis

Audrey HEMADOU[1], Jeanny LAROCHE-TRAINEAU[†1], Ségolène ANTOINE[†1], Philippe MONDON[2], Alexandre FONTAYNE[2], Yannick LE PRIOL[3], Stéphane CLAVEROL[4], Stéphane SANCHEZ[1], Martine CERUTTI[5], Florence OTTONES[1], Gisèle CLOFENT-SANCHEZ[†1] and Marie-Josée JACOBIN-VALAT[†*1]

[†]Equivalent position of authors

[1] CRMSB, UMR5536 CNRS, INSB, Bordeaux, 33076, France

[2] LFB Biotechnologies, department of biotherapeutic engineering Lille, 59000, France

[3] Elsevier Masson SAS, Elsevier RD solutions, Issy les Moulineaux, 92130, France

[4] CGFB, Proteome pole, Bordeaux, 33076, France

[5] UPS 3044, CNRS, Saint-Christol-Lès-Alès, France

1 Supplementary Methods

1.1 S1. ScFv sequencing

Sanger sequencing was performed using primers specific to flanking regions in the phagemide (5'-TGCAAATTCTATTTCAAGGAGAC-3' and 5'-AGAATCATCAGATAAAGTAATCC-3'). Antibody gene fragments were analysed by IMGT/V-QUEST database (www.imgt.org/IMGT_vquest/vquest) for V germline determination and CDR3 analyses.

1.2 S2. Western blot analyses of scFv displayed on phages

After migration of the scFv-phages on 4-15% SDS-PAGE followed by western blotting, the scFv expression was detected using as primary antibody, a murine anti-pIII protein antibody (Mobitec, Ozyme, France) at 1 µg/mL in MPBS 0.5% as described earlier[12]. Secondary anti mouse HRP (1:1000) antibody was added and incubated at RT for 2 h. Each step was followed by extensive washing and chemoluminescent signal was developed with ECL reagent (Thermofisher, France).

1.3 S3. Immunohistochemical analysis on NZW rabbit sections

Clones screened positively by flow cytometry were evaluated for binding to paraffin-embedded atheromatous tissue samples prepared from the same atheromatous specimen. Briefly, after a retrieval step in retrieval buffer (TRIS 10mM, EDTA 1mM, 0.05% Tween 20, pH 9), deparaffinized tissue sections were incubated with scFv-phages overnight at 4°C, washed with PBS BSA 2%. Sections were then incubated with mouse anti-pVIII protein antibody diluted at 1:200 (Abcam, France) for 1h and then with envision HRP system anti-mouse. Binding was detected using DAB system (Dako, France).

1.4 S4. *In vivo* phage-display

The elution protocols of endothelial, intra-tissular and intra-cellular scFv fractions were performed according to Deramchia et al[7], with minor modifications. The endothelium cell surface-bound scFv-phage fraction (F1 fraction) was eluted with 500 µL of 0.1 M glycine-HCl, pH 2.2 and immediately neutralized with 15 µL of 2.5 M Tris-HCl, pH 8. The elution procedure was repeated, the different samples were pooled, a protease-inhibitor cocktail was added (ThermoScientific) and the fraction was stored at 4 °C prior bacterial infection.

In order to elute intra-tissular scFv-phage fraction (F2 fraction), the aortic tissue was incubated with 900 µL of PBS (Ca^{2+}, Mg^{2+} free) containing 2,000 U/mL of collagenase type II (Gibco) adjusted to 1 mL with 2.5% Trypsin-EDTA (Eurobio) and scratched using a glass slide to facilitate the tissue dissociation. Cycles of 20 s Polytron homogenizer (Ultraturax TP-20, Kinematica) were then performed at 4 °C to

obtain a homogeneous solution. The homogenate was then incubated at 37 °C for 30 min with punctual vortexing and finally centrifuged for 10 minutes at 1,000 g to remove insoluble material. This preparation was homogenized 2 times more following the same procedure. After each centrifugation, supernatants containing eluted scFv-phages were collected and pooled in a clean tube in presence of a protease-inhibitor cocktail.

To access the internalized scFv-phage fraction (F3 fraction), the insoluble material was incubated with 500 µL of 0.1 M TEA (Tetraethylammonium chloride) (Sigma-Aldrich), pH 11.5 for 5 min at RT and then vigorously vortexed for another 5 min. Samples were neutralized by addition of 150 µL of 1M Tris-HCl, pH 7.4. After centrifugation at 1,000 g for 10 min, the supernatant was collected.

2 Supplementary Figure legends

Figure S1. Phage enrichment after three rounds of *in vivo* phage display biopanning. P1: first round of biopanning, P2: second round and P3: third round. E1: aortic arch and thoracic area, E2: abraded renal and abdominal areas. F1: endothelial fraction, F2: intra-tissular fraction, F3: intra-cellular fraction.

Figure S2. Evaluation of the coupling rate of balloon injured aortas of hypercholesterolemic NZW rabbit (BIAHR) proteins on beads. A range of BIAHR proteins (40 to 8µg) was used to evaluate the coupling rate of proteins on beads by SDS-PAGE. Image J analysis allowed to estimate the coupling rate at 80%.

Figure S3. Schematic representation of conditions tested for the implementation of the flow cytometry strategy. Four conditions have been carried out to determine the optimum parameters for a high-throughput screening of scFv-phages from the 3 rd round of selection by *in vivo* phage display.

Figure S4. ScFv-phage production analysed by SDS-PAGE. Example of eight scFv-phage clones produced after infection of recombinant bacteria with hyperphage. Proteins were separated on 4-15% Bis acrylamide gel. The pIII protein fragment was detected by mouse anti-pIII protein antibody and HRP anti mouse antibody. SuperSignal west pico chemiluminescent substrate was used for staining the membrane. Images were analysed by Snapgene software. The white lineage corresponds to a crop of the western blot membrane.

Figure S5: (A): Workflow of the strategy for the screening and selection of scFv-phage clones derived from *in vivo* phage display biopanning of a fully human library in the atherosclerotic NZW rabbit model. (B): Mass spectrometry on extracted atheromatous proteins used for flow cytometry screening, highlighting potential biomarkers.

3 Supplementary figures

Figure S1:

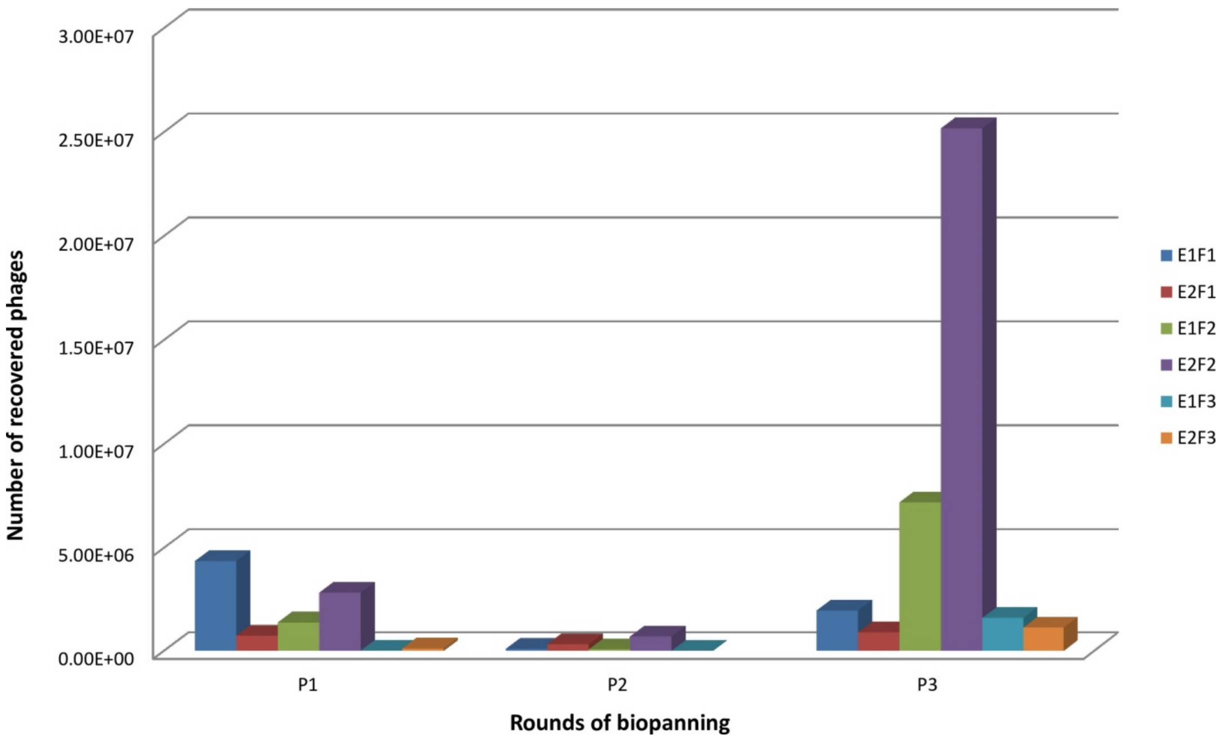

Figure S2 :

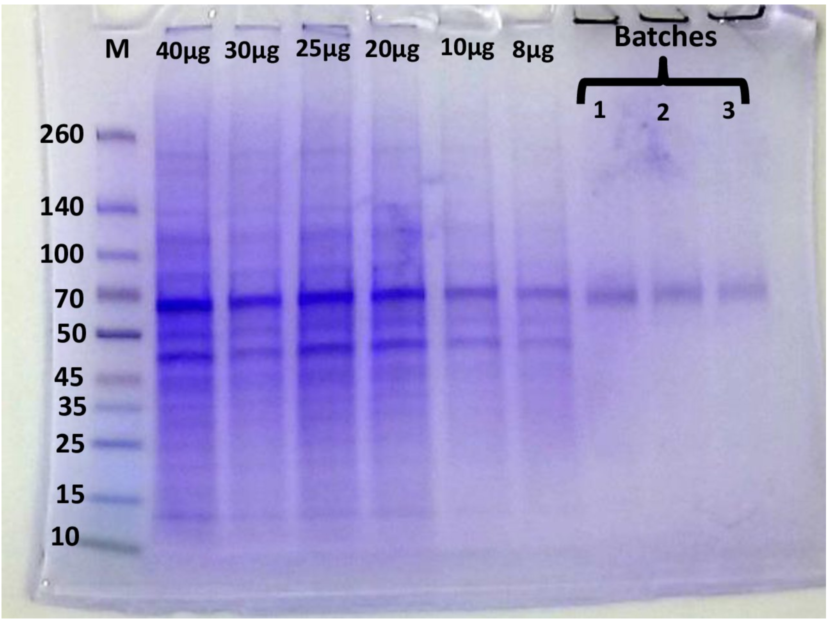

Figure S3 :

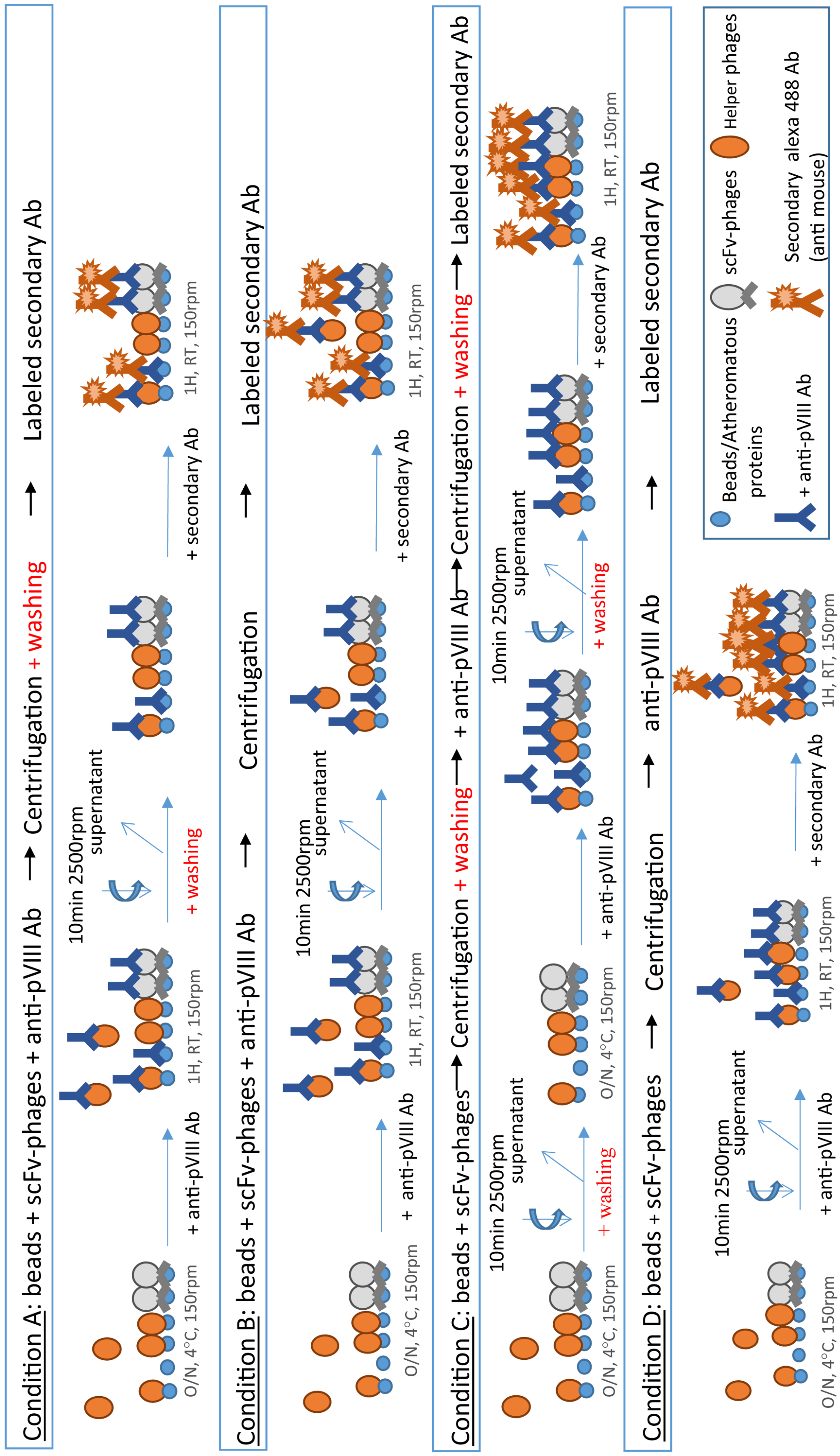
Condition A: beads + scFv-phages + anti-pVIII Ab → Centrifugation + washing → Labeled secondary Ab
O/N, 4°C, 150rpm
+ anti-pVIII Ab
1H, RT, 150rpm
10min 2500rpm
supernatant
+ washing
+ secondary Ab
1H, RT, 150rpm
Condition B: beads + scFv-phages + anti-pVIII Ab → Centrifugation → Labeled secondary Ab
O/N, 4°C, 150rpm
+ anti-pVIII Ab
1H, RT, 150rpm
10min 2500rpm
supernatant
+ secondary Ab
1H, RT, 150rpm
Condition C: beads + scFv-phages → Centrifugation + washing → + anti-pVIII Ab → Centrifugation + washing → Labeled secondary Ab
O/N, 4°C, 150rpm
10min 2500rpm
supernatant
+ washing
O/N, 4°C, 150rpm
+ anti-pVIII Ab
10min 2500rpm
supernatant
+ washing
+ secondary Ab
1H, RT, 150rpm
Condition D: beads + scFv-phages → Centrifugation → anti-pVIII Ab → Labeled secondary Ab
O/N, 4°C, 150rpm
10min 2500rpm
supernatant
+ anti-pVIII Ab
1H, RT, 150rpm
+ secondary Ab
1H, RT, 150rpm
Beads/Atheromatous proteins
scFv-phages
Helper phages
+ anti-pVIII Ab
Secondary alexa 488 Ab (anti mouse)

Figure S4 :

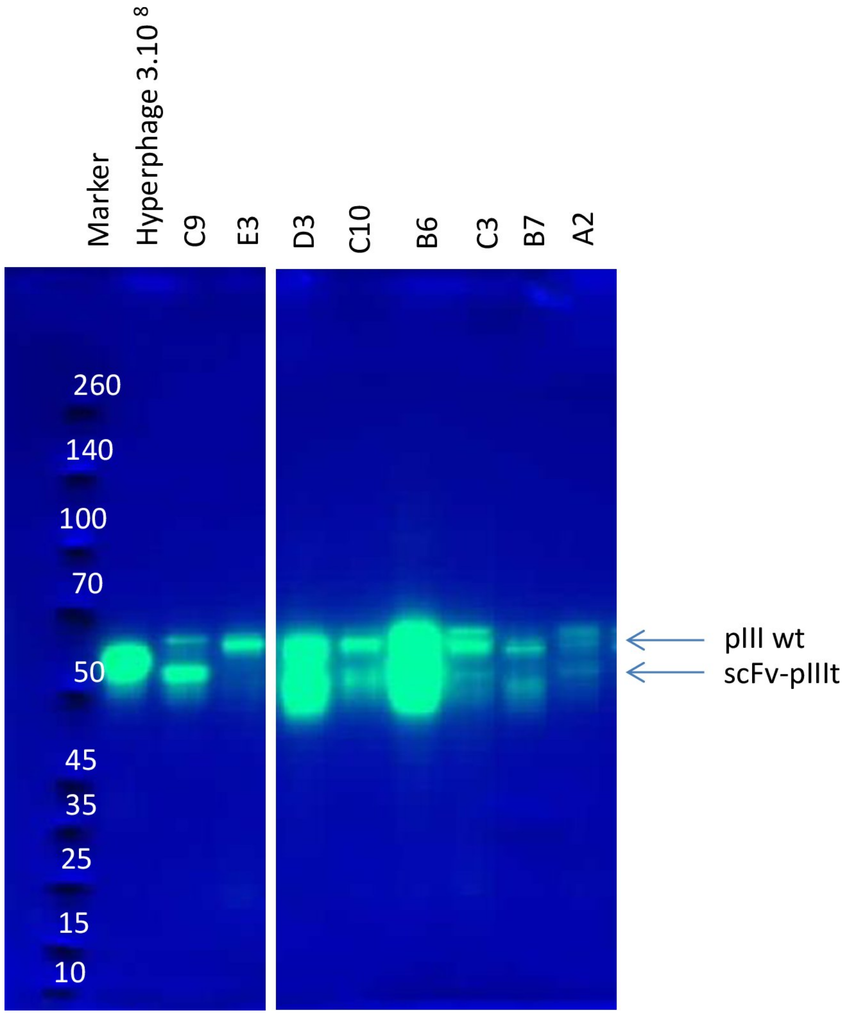
Marker
Hyperphage 3.10 8
C9
E3
D3
C10
B6
C3
B7
A2
260
140
100
70
50
45
35
25
15
10
pIII wt
scFv-pIIIt

Figure S5:

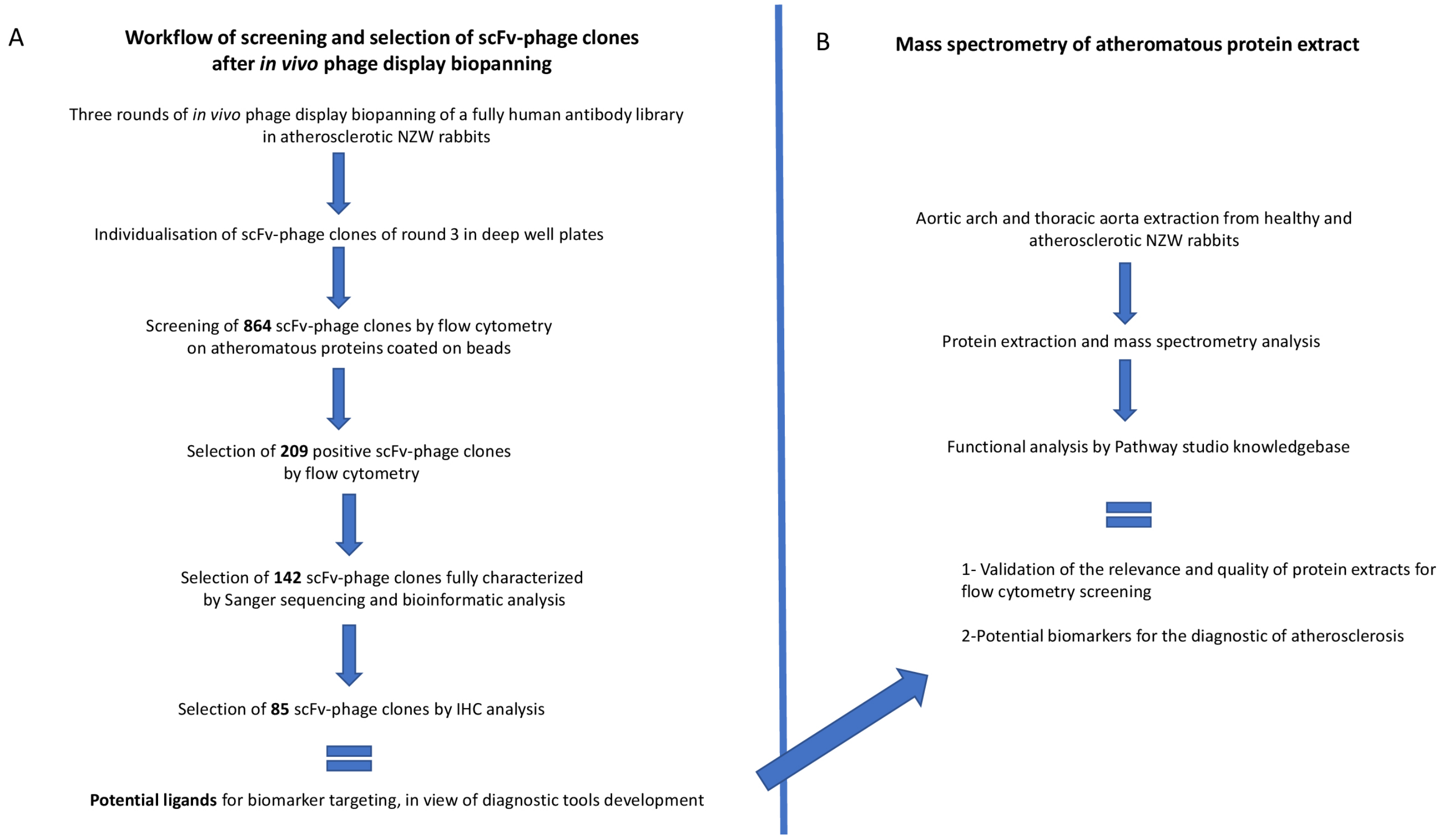

4 Supplementary Tables

ratio BIAHR/H proteins	Description
7898.19	Fibulin-7 OS=Homo sapiens GN=FBLN7 PE=2 SV=1 - [FBLN7_HUMAN]
3478.34	Lysophosphatidylcholine acyltransferase 2 OS=Homo sapiens GN=LPCAT2 PE=1 SV=1 - [PCAT2_HUMAN]
253.68	Fatty acid-binding protein, brain OS=Homo sapiens GN=FABP7 PE=1 SV=3 - [FABP7_HUMAN]
234.64	Beta-galactosidase-1-like protein OS=Homo sapiens GN=GLB1L PE=2 SV=1 - [GLB1L_HUMAN]
215.31	Receptor-type tyrosine-protein phosphatase C OS=Homo sapiens GN=PTPRC PE=1 SV=2 - [PTPRC_HUMAN]
202.46	Fatty acid-binding protein, epidermal OS=Homo sapiens GN=FABP5 PE=1 SV=3 - [FABP5_HUMAN]
182.25	Gamma-enolase OS=Homo sapiens GN=ENO2 PE=1 SV=3 - [ENOG_HUMAN]
147.57	Polyadenylate-binding protein 4 OS=Homo sapiens GN=PABPC4 PE=1 SV=1 - [PABP4_HUMAN]
147.16	Integrin beta-2 OS=Homo sapiens GN=ITGB2 PE=1 SV=2 - [ITB2_HUMAN]
131.89	Perilipin-2 OS=Homo sapiens GN=PLIN2 PE=1 SV=2 - [PLIN2_HUMAN]
131.69	Hypothetical drug-resistance-associated protein OS=Homo sapiens PE=4 SV=1 - [Q86YX8_HUMAN]
122.44	Carabin OS=Homo sapiens GN=TBC1D10C PE=1 SV=1 - [TB10C_HUMAN]
109.92	Alpha-actinin-1 OS=Homo sapiens GN=ACTN1 PE=1 SV=2 - [ACTN1_HUMAN]

106.83	Serum albumin OS=Homo sapiens GN=ALB PE=1 SV=2 - [ALBU_HUMAN]
105.52	Clusterin OS=Homo sapiens GN=CLU PE=1 SV=1 - [CLUS_HUMAN]
101.81	Apoptosis regulator BAX OS=Homo sapiens GN=BAX PE=1 SV=1 - [BAX_HUMAN]
79.10	Tetranectin OS=Homo sapiens GN=CLEC3B PE=1 SV=3 - [TETN_HUMAN]
76.12	Transmembrane channel-like protein 2 OS=Homo sapiens GN=TMC2 PE=2 SV=3 - [TMC2_HUMAN]
71.83	Cartilage oligomeric matrix protein OS=Homo sapiens GN=COMP PE=1 SV=2 - [COMP_HUMAN]
64.54	Ras-related C3 botulinum toxin substrate 2 OS=Homo sapiens GN=RAC2 PE=1 SV=1 - [RAC2_HUMAN]
62.99	Probable ATP-dependent RNA helicase DDX6 OS=Homo sapiens GN=DDX6 PE=1 SV=2 - [DDX6_HUMAN]
62.63	Syntenin-1 OS=Homo sapiens GN=SDCBP PE=1 SV=1 - [SDCB1_HUMAN]
57.28	Beta-glucuronidase OS=Homo sapiens GN=GUSB PE=1 SV=2 - [BGLR_HUMAN]
54.16	Serine protease HTRA1 OS=Homo sapiens GN=HTRA1 PE=1 SV=1 - [HTRA1_HUMAN]
50.26	Macrophage-capping protein OS=Homo sapiens GN=CAPG PE=1 SV=2 - [CAPG_HUMAN]
48.10	Histamine N-methyltransferase OS=Homo sapiens GN=HNMT PE=1 SV=1 - [HNMT_HUMAN]
45.74	Serum paraoxonase/lactonase 3 OS=Homo sapiens GN=PON3 PE=1 SV=3 - [PON3_HUMAN]
45.43	Purine nucleoside phosphorylase OS=Homo sapiens GN=PNP PE=1 SV=2 - [PNPH_HUMAN]
42.24	Inter-alpha-trypsin inhibitor heavy chain H1 OS=Homo sapiens GN=ITIH1 PE=1 SV=3 - [ITIH1_HUMAN]
41.21	Ig gamma-1 chain C region OS=Homo sapiens GN=IGHG1 PE=1 SV=1 - [IGHG1_HUMAN]
38.53	Alpha-N-acetylgalactosaminidase OS=Homo sapiens GN=NAGA PE=1 SV=2 - [NAGAB_HUMAN]
38.38	Coiled-coil domain-containing protein 80 OS=Homo sapiens GN=CCDC80 PE=1 SV=1 - [CCD80_HUMAN]
38.05	Vitamin D-binding protein OS=Homo sapiens GN=GC PE=1 SV=1 - [VTDB_HUMAN]
37.69	Plasminogen OS=Homo sapiens GN=PLG PE=1 SV=2 - [PLMN_HUMAN]
35.82	Protein TANC1 OS=Homo sapiens GN=TANC1 PE=1 SV=3 - [TANC1_HUMAN]
31.59	Complement component C7 OS=Homo sapiens GN=C7 PE=1 SV=2 - [CO7_HUMAN]
31.15	UPF0554 protein C2orf43 OS=Homo sapiens GN=C2orf43 PE=1 SV=1 - [CB043_HUMAN]
26.66	Histidine-rich glycoprotein OS=Homo sapiens GN=HRG PE=1 SV=1 - [HRG_HUMAN]
25.58	60S ribosomal protein L35a OS=Homo sapiens GN=RPL35A PE=1 SV=2 - [RL35A_HUMAN]
22.74	Plastin-2 OS=Homo sapiens GN=LCP1 PE=1 SV=6 - [PLSL_HUMAN]
21.94	Adipocyte plasma membrane-associated protein OS=Homo sapiens GN=APMAP PE=1 SV=2 - [APMAP_HUMAN]
20.85	Alpha-2-macroglobulin OS=Homo sapiens GN=A2M PE=1 SV=3 - [A2MG_HUMAN]

20.41	Retinal dehydrogenase 2 OS=Homo sapiens GN=ALDH1A2 PE=1 SV=3 - [AL1A2_HUMAN]
19.84	Complement C4-A OS=Homo sapiens GN=C4A PE=1 SV=2 - [CO4A_HUMAN]
19.81	Insulin-like growth factor-binding protein 7 OS=Homo sapiens GN=IGFBP7 PE=1 SV=1 - [IBP7_HUMAN]
19.43	Peroxisomal acyl-coenzyme A oxidase 1 OS=Homo sapiens GN=ACOX1 PE=1 SV=3 - [ACOX1_HUMAN]
19.42	Ferritin heavy chain OS=Homo sapiens GN=FTH1 PE=1 SV=2 - [FRIH_HUMAN]
19.39	Lactotransferrin OS=Homo sapiens GN=LTF PE=1 SV=6 - [TRFL_HUMAN]
19.36	Glypican-6 OS=Homo sapiens GN=GPC6 PE=1 SV=1 - [GPC6_HUMAN]
18.69	Pro-cathepsin H OS=Homo sapiens GN=CTSH PE=1 SV=4 - [CATH_HUMAN]
18.53	Complement C5 OS=Homo sapiens GN=C5 PE=1 SV=4 - [CO5_HUMAN]
18.06	Serotransferrin OS=Homo sapiens GN=TF PE=1 SV=3 - [TRFE_HUMAN]
17.91	60S ribosomal protein L36a OS=Homo sapiens GN=RPL36A PE=1 SV=2 - [RL36A_HUMAN]
17.69	Galectin-3 OS=Homo sapiens GN=LGALS3 PE=1 SV=5 - [LEG3_HUMAN]
17.68	Protein flightless-1 homolog OS=Homo sapiens GN=FLII PE=1 SV=2 - [FLII_HUMAN]
17.54	2,4-dienoyl-CoA reductase, mitochondrial OS=Homo sapiens GN=DECR1 PE=1 SV=1 - [DECR_HUMAN]
17.33	Apolipoprotein A-I OS=Homo sapiens GN=APOA1 PE=1 SV=1 - [APOA1_HUMAN]
17,11	Beta-hexosaminidase subunit alpha OS=Homo sapiens GN=HEXA PE=1 SV=2 - [HEXA_HUMAN]
16.78	C-terminal-binding protein 1 OS=Homo sapiens GN=CTBP1 PE=1 SV=2 - [CTBP1_HUMAN]
16.76	Coronin-1A OS=Homo sapiens GN=CORO1A PE=1 SV=4 - [COR1A_HUMAN]
16.49	Complement component C6 OS=Homo sapiens GN=C6 PE=1 SV=3 - [CO6_HUMAN]
15.75	3-hydroxybutyrate dehydrogenase type 2 OS=Homo sapiens GN=BDH2 PE=1 SV=2 - [BDH2_HUMAN]
15.64	Twinfilin-1 OS=Homo sapiens GN=TWF1 PE=1 SV=3 - [TWF1_HUMAN]
15.59	Glycylpeptide N-tetradecanoyltransferase 1 OS=Homo sapiens GN=NMT1 PE=1 SV=2 - [NMT1_HUMAN]
14.75	Lysosome membrane protein 2 OS=Homo sapiens GN=SCARB2 PE=1 SV=2 - [SCRB2_HUMAN]
14.71	Galectin-3-binding protein OS=Homo sapiens GN=LGALS3BP PE=1 SV=1 - [LG3BP_HUMAN]
14.53	Apolipoprotein B-100 OS=Homo sapiens GN=APOB PE=1 SV=2 - [APOB_HUMAN]
14.48	Ig kappa chain V-I region Roy OS=Homo sapiens PE=1 SV=1 - [KV116_HUMAN]
14.42	Collagen triple helix repeat-containing protein 1 OS=Homo sapiens GN=CTHRC1 PE=1 SV=1 - [CTHR1_HUMAN]
14.08	Cathepsin D OS=Homo sapiens GN=CTSD PE=1 SV=1 - [CATD_HUMAN]
14.06	Fibrinogen gamma chain OS=Homo sapiens GN=FGG PE=1 SV=3 - [FIBG_HUMAN]
13.69	Integrin beta-3 OS=Homo sapiens GN=ITGB3 PE=1 SV=2 - [ITB3_HUMAN]
13.44	Hemopexin OS=Homo sapiens GN=HPX PE=1 SV=2 - [HEMO_HUMAN]
13.34	Glia-derived nexin OS=Homo sapiens GN=SERPINE2 PE=1 SV=1 - [GDN_HUMAN]

13.02	Flotillin-2 OS=Homo sapiens GN=FLOT2 PE=1 SV=2 - [FLOT2_HUMAN]
12.96	Eukaryotic translation initiation factor 4 gamma 1 OS=Homo sapiens GN=EIF4G1 PE=2 SV=1 - [E7EX73_HUMAN]
12.75	Protein FAM49B OS=Homo sapiens GN=FAM49B PE=1 SV=1 - [FA49B_HUMAN]
12.56	EH domain-containing protein 4 OS=Homo sapiens GN=EHD4 PE=1 SV=1 - [EHD4_HUMAN]
12.42	Beta-galactosidase OS=Homo sapiens GN=GLB1 PE=1 SV=2 - [BGAL_HUMAN]
11.96	Protein S100-A4 OS=Homo sapiens GN=S100A4 PE=1 SV=1 - [S10A4_HUMAN]
11.78	Ig alpha-2 chain C region OS=Homo sapiens GN=IGHA2 PE=1 SV=3 - [IGHA2_HUMAN]
11.40	Aldehyde oxidase OS=Homo sapiens GN=AOX1 PE=1 SV=2 - [AOXA_HUMAN]
11.37	Alanine--tRNA ligase, cytoplasmic OS=Homo sapiens GN=AARS PE=1 SV=2 - [SYAC_HUMAN]
11.26	Fibrinogen beta chain OS=Homo sapiens GN=FGB PE=1 SV=2 - [FIBB_HUMAN]
11.18	Soluble scavenger receptor cysteine-rich domain-containing protein SSC5D OS=Homo sapiens GN=SSC5D PE=2 SV=3 - [SRCRL_HUMAN]
11.06	Twinfilin-2 OS=Homo sapiens GN=TWF2 PE=1 SV=2 - [TWF2_HUMAN]
10.91	Lactadherin OS=Homo sapiens GN=MFGE8 PE=1 SV=2 - [MFGM_HUMAN]
10.83	Transmembrane protein 245 OS=Homo sapiens GN=TMEM245 PE=1 SV=2 - [TM245_HUMAN]
10.82	Apolipoprotein E OS=Homo sapiens GN=APOE PE=1 SV=1 - [APOE_HUMAN]
10.63	b(0,+)-type amino acid transporter 1 OS=Homo sapiens GN=SLC7A9 PE=4 SV=1 - [K7EKD0_HUMAN]
10.55	Glia maturation factor gamma OS=Homo sapiens GN=GMFG PE=1 SV=1 - [GMFG_HUMAN]
10.13	Haptoglobin OS=Homo sapiens GN=HP PE=1 SV=1 - [HPT_HUMAN]
9.94	Fibulin-1 OS=Homo sapiens GN=FBLN1 PE=1 SV=4 - [FBLN1_HUMAN]
9.85	Dehydrogenase/reductase SDR family member 4 OS=Homo sapiens GN=DHRS4 PE=1 SV=3 - [DHRS4_HUMAN]
9.29	2-oxoisovalerate dehydrogenase subunit alpha, mitochondrial OS=Homo sapiens GN=BCKDHA PE=1 SV=2 - [ODBA_HUMAN]
9.01	Collagen alpha-1(I) chain OS=Homo sapiens GN=COL1A1 PE=1 SV=5 - [CO1A1_HUMAN]
8.97	Ceruloplasmin OS=Homo sapiens GN=CP PE=1 SV=1 - [CERU_HUMAN]
8.95	Disabled homolog 2 OS=Homo sapiens GN=DAB2 PE=1 SV=3 - [DAB2_HUMAN]
8.64	Xanthine dehydrogenase/oxidase OS=Homo sapiens GN=XDH PE=1 SV=4 - [XDH_HUMAN]
8.40	V-type proton ATPase subunit E 1 OS=Homo sapiens GN=ATP6V1E1 PE=1 SV=1 - [VATE1_HUMAN]
8.37	Carbonic anhydrase 2 OS=Homo sapiens GN=CA2 PE=1 SV=2 - [CAH2_HUMAN]

Table S2. Proteins over-represented in balloon injured aortas of hypercholesterolemic NZW rabbit (BIAHR) extract versus Healthy (H) extracts. The choice of the relevant proteins in the LS-MS / MS studies is carried out according to 3 main criteria: (1) a number of identified peptides in human database greater than 2, (2) the calcul of ratios normalized protein abundance between BIAHR and H extracts, (3) a minimum BIAHR / H ratio threshold of 8.

Masterblock	Fractions	Selected clones	Clones with an in frame scFv sequence	Nb of VH clonotypes	Nb of VL clonotypes
PL19	E1F1	12	8	7	8
PL20	E2F1	27	17	15	17
PL21	E1F2	15	11	10	11
PL22	E1F2	36	19	19	18
PL23	E2F2	61	45	40	41
PL24	E2F2	10	9	9	9
PL25	E1F3	13	11	11	10
PL26	E1F3	18	10	10	10
PL27	E2F3	17	12	10	10

Table S3. Sanger Sequencing and IMGT-VQUEST analysis performed on 209 clones selected by flow cytometry. Among these, 68% of them have a complete sequence with 42% without stop codons. Analyses of the germlines and CDR3H and CDR3L junctions of each scFv showed a high diversity among the clones.

Additional Information – supplementary data -

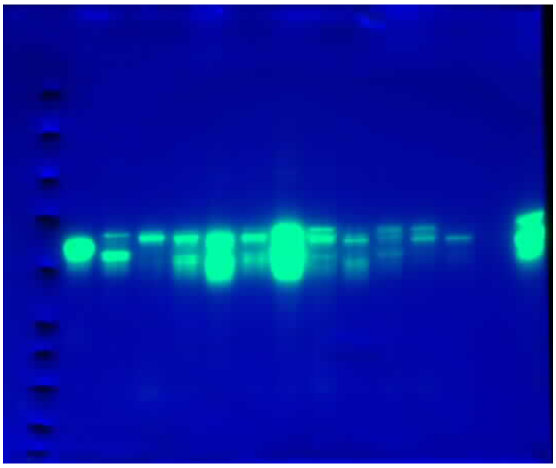

Supplement: Supplementary file 2 — Supplementary information 2 [file 41598_2018_33382_MOESM2_ESM.pdf]
